# Leveraging LiDAR-Based Simulations to Quantify the Complexity of the Static Environment for Autonomous Vehicles in Rural Settings

**DOI:** 10.3390/s24020452

**Published:** 2024-01-11

**Authors:** Mohamed Abohassan, Karim El-Basyouny

**Affiliations:** Department of Civil and Environmental Engineering, Faculty of Engineering, University of Alberta, Edmonton, AB T6G 1H9, Canada; basyouny@ualberta.ca

**Keywords:** autonomous vehicles, virtual simulations, LiDAR data, digital twins, data processing, wildlife–vehicle collisions

## Abstract

This paper uses virtual simulations to examine the interaction between autonomous vehicles (AVs) and their surrounding environment. A framework was developed to estimate the environment’s complexity by calculating the real-time data processing requirements for AVs to navigate effectively. The VISTA simulator was used to synthesize viewpoints to replicate the captured environment accurately. With an emphasis on static physical features, roadways were dissected into relevant road features (RRFs) and full environment (FE) to study the impact of roadside features on the scene complexity and demonstrate the gravity of wildlife–vehicle collisions (WVCs) on AVs. The results indicate that roadside features substantially increase environmental complexity by up to 400%. Increasing a single lane to the road was observed to increase the processing requirements by 12.3–16.5%. Crest vertical curves decrease data rates due to occlusion challenges, with a reported average of 4.2% data loss, while sag curves can increase the complexity by 7%. In horizontal curves, roadside occlusion contributed to severe loss in road information, leading to a decrease in data rate requirements by as much as 19%. As for weather conditions, heavy rain increased the AV’s processing demands by a staggering 240% when compared to normal weather conditions. AV developers and government agencies can exploit the findings of this study to better tailor AV designs and meet the necessary infrastructure requirements.

## 1. Introduction

One of the challenges that autonomous vehicles (AVs) face is the large amount of data inundating the onboard computer from its sensors, which is beyond the real-time processing capabilities of current AV models [1,2]. LiDAR, for example, can collect more than 200,000 points per second, depending on the model [3]. Hence, AVs strategically employ preprocessing techniques during the environment perception phase to streamline computational complexity [4]. This intelligent data prioritization mechanism involves pruning out extraneous road features. Relevant aspects, such as road surfaces, including lanes and boundaries, traffic signs and signals, as well as the presence of vehicles and pedestrians, are the primary focus of this process [5,6]. By selectively prioritizing these crucial elements, AVs enhance their efficiency and decision-making capabilities. Data compression techniques are also developed to improve real-time performance [7].

At present, AVs’ operations have been limited to testing and piloting initiatives; their activities have been confined to specific regions where road conditions are known, and the environments are reasonably predictable [8]. The current data acquired from laboratory-based approaches and typical test tracks are insufficient to assess AVs’ safety. Some estimates believe that, for AVs to achieve a 95% confidence that their failure rates are better than human driver failures, they are required to drive an excess of 275 million miles [9], and the most optimistic projections expect that this feat can be achieved in 84 years [10].

For domains where safety is of the utmost importance, such as AVs, simulation-based approaches are indispensable for developers and researchers [11]. Such approaches can offer a safe context to conduct necessary large-scale testing and validations via the digitization of the environment [12]. In addition, since physical testing for AVs is generally banned inside most cities, the only real-life substitute would be time-consuming and expensive field tests, which are also hard to reproduce and often seen as inconvenient [13].

The high-fidelity 3D point cloud data collected from LiDAR is particularly effective for this purpose due to its millimeter-level accuracy [14]. This LiDAR data can be used to create digital twins [15] of the physical environment and opens the door for risk-free simulation environments, especially since the sought-after safety levels cannot be achieved until billions of kilometers of testing have been taken by the AVs under different weather conditions [16]. Mobile laser scanning data (MLS) has been widely recognized to be the more expedient form of LiDAR data as it is known for the high level of detail for its outputs, cheap cost of operation, high point density, and the ability to scan below bridges and inside tunnels [17].

This study was performed on 34 km of two-way, two-lane highways in rural environments in Alberta, Canada, to better isolate the different physical road features and facilitate the investigation of their influence on the performance of the AVs. For instance, occlusion handling for AVs is one of the most challenging tasks [18]; hence, it is essential to study its risks on AV operations. Furthermore, since LiDAR is the most preferred sensor in the majority of driverless vehicles [19], it has been employed as the primary visionary sensor in the simulations.

The main contribution of this work is the development of a novel framework that can estimate the AV environment complexity using a transformative function that can translate the dynamicity of the surrounding environment [20] along with the AV sensor capabilities and the influence of weather conditions into interpretable data rate values via lidar-based simulations using the Vista simulator [21]. This framework excels in detecting the critical areas within the physical infrastructure for AVs while also quantifying their complexity levels which is an unprecedented feat in the literature.

The rest of this article is organized as follows: A thorough literature review conducted to identify the existing gaps is presented in Section 2. Subsequently, the adopted methodology will be outlined in Section 3, leading to the presentation of the numerical analysis results in Section 4. Following this, a comprehensive discussion will ensue in Section 5. Lastly, the conclusions of this work, along with the research limitations and future research prospects, are outlined in Section 6.

## 2. Literature Review

### 2.1. Autonomous Vehicles

The ongoing developments in autonomous driving prompted the Society of Automotive Engineers (SAE) to define six levels of driving automation [22]. The real challenge starts with level 3 conditional automation, where the driver’s attention is only called to respond to emergencies, whereas, during normal driving conditions, the human is not required to control the vehicle. Automated driving systems (ADS), in general, have their restrictions, defined as operational design domain (ODD) [23]. Human supervision is not required for levels 4 and 5 of automation. The only difference is that level 4 is active in some ODDs since it needs the support of detailed maps and the existence of certain types of infrastructures. If these conditions are unmet, the vehicles automatically park themselves to stop the trip as a part of a fail-safe system [24]. On the other hand, level 5 is designed to have full automation without any human intervention.

Currently, some AV models indeed operate on level 4 autonomous driving [25]. However, due to the lack of adequate infrastructure and supporting legislation, their deployment has been restricted to a few small regions with urban environments with a speed limit of only 50 kph [25]. Such regulations prompted these models to be used primarily for ridesharing purposes. WAYMO, NAVYA, and Magna are among the presently available level 4 AVs [25]. Numerous companies, including Audi, Uber, WAYMO, and Tesla, have openly acknowledged their ongoing efforts to test level 5 autonomous vehicles for future public use. However, no level 5 autonomous vehicle has been released for commercial use [25].

Several challenges impede the realization and deployment of fully autonomous vehicles. Such challenges include technological, safety, ethical, and political aspects [26]. A significant technological hurdle arises from the immense data influx into the AV’s onboard computer, primarily from sensors like LiDAR, which complicates real-time data processing and subsequently impacts the vehicle’s efficiency and safety. The literature shows that leveraging parallel computations is a highly effective strategy for addressing real-time processing challenges associated with large datasets. For instance, [27] developed an optimized algorithm using the OpenMP technology to expedite the processing time of determining the position of the LiDAR by eight times. This is especially relevant as computing system architectures are currently experiencing substantial advancements and are positioned to become even more sophisticated in the future [27].

The concept of vehicle-to-anything communication (V2X) also holds promise as a solution to this challenge, especially since it has seen rapid advancements in recent years. For instance, in [28], smart nodes were designed to help optimize communications of the vehicles with the infrastructure by learning the environment with its variable scenarios and predicting the optimal minimum contention window (MCW) by training DQN models. Nevertheless, the current infrastructure cannot support this technology yet, and existing networks lack the robustness needed to support the anticipated high volume of data exchange [25]. Additionally, safeguarding the privacy of the extensive data collected by AVs is a vital concern [29].

Furthermore, the correct and timely response to the surprising loss of control incidents like skidding is not on par with human reactions [30]. Uncrewed autonomous vehicles face a significant challenge in matching or improving upon human factors, such as ethical decision making on the road. While these vehicles excel at following traffic rules and safe navigation, they lack the “human touch” needed to make moral decisions in complex situations that involve human emotions, morals, and judgment [26]. This lack raises concerns about potential biases in the algorithms or AI used in AVs [26], especially when it is believed that the public will switch from crewed to uncrewed vehicles only after they understand the ethical principles that the AVs follow [8]. A different facet that impedes the progress of AVs is the current policies and regulations. Liability, in particular, emerges as a pivotal concern for the widespread adoption of AVs. Currently, drivers are typically liable for any car-involved collisions [31]. However, determining the primary responsible party becomes rather unclear in the context of accidents involving uncrewed vehicles [32].

### 2.2. AV Simulations on LiDAR Data

Achieving a meticulous replication of the intricate physical road infrastructure and an exacting simulation of the physics involved in sensing processes is key to bridging the substantial divide between theoretical simulations and real-world applications [19]. Closing this gap not only refines the development of AV technologies but also augments their reliability, safety, and efficiency. Broadly speaking, LiDAR data can either be real or synthetic, and it is worth noting that the use of synthetic LiDAR data in simulations is yet to be on par with the use of realistic data as they were shown to have lesser accuracies in capturing the intricacies of the real-world environment and were generally not as diverse [33].

Neuhaus et al. [34] utilized the 3D point cloud data captured by the Velodyne HDL-64E LiDAR sensor in assessing autonomous navigations in unstructured environments. Drivable areas were analyzed using an innovative algorithm that evaluates local terrain roughness, enhancing the precision of autonomous vehicle path planning. Furthermore, Manivasagam et al. [19] leveraged real data collected by their self-driving fleet in diverse cities to enhance simulation realism. They curated an extensive catalog of 3D static maps and dynamic objects from real-world situations to devise an innovative simulator that integrates physics-based and learning-based approaches. Li et al. [35] proposed augmented autonomous driving simulation (AADS) by combining LiDAR and cameras to scan real street scenes. In contrast to traditional approaches, their method offers more scalability and realism. Likewise, Fang et al. [36] utilized MLS data to create a virtual environment that can directly reflect the complexity and diversity of the real-world geometry. Then, by applying CAD models to capture the obstacles’ poses, such information was incorporated into the virtual environment to enrich it and enhance the AV simulations. This method demonstrated that a combination of real and simulated data can attain over 95% accuracy in the simulations.

Contrastively, several other works have used synthetic LiDAR data in their AV simulations since they do not require the same heavy manual annotation work as the scanned LiDAR data. Hence, they promise to streamline and increase the efficiency of AV simulations [35]. For instance, the authors in [37] extracted their synthetic LiDAR annotated datasets from the famous computer game GTA-V, where they simulated a virtual scanning vehicle within the game’s environment to capture realistic driving scenes. On the other hand, Wang et al. [38] developed a framework that simulated LiDAR sensors in the CARLA [11] autonomous urban driving simulator to generate synthetic LiDAR data. Their approach was inspired by the notable achievements of deep learning in 3D data analysis. This method demonstrated that incorporating synthetic data significantly improves the performance and accuracy of AVs in simulation environments.

### 2.3. Quantifying the Complexity of the AV Environment

The onboard computer equipped by the AVs, which is required to operate in real- time, has the onus of perceiving the environment surrounding the AV, processing the incoming information, and making the most apt decisions to ensure the safe operations of the ego vehicle. The traffic environment poses a big challenge for autonomous systems as they are typically open, non-deterministic, hard to predict, and dynamic [20]. Identifying the complex situations is essential in advancing AVs’ safety, which would expedite their mass adoption.

Wang et al. [39] proposed a modeling and assessment framework that can quantify the complexity of the AV’s environment. Their approach involved establishing fundamental and additional environmental complexity models that systematically evaluate four key environmental aspects: road conditions, traffic features, weather conditions, and potential interferences. Based on experts’ judgment, the overall environment complexity can be calculated using a preset scoring system for the different environment features. The analytic hierarchy method (AHM) determines the relation between different attributes. A weighting scheme based on subjective and objective considerations is implemented to calculate the overall complexity of the environment. A similar, automated framework that bases its measured complexity on the road type, scene type, challenging conditions, and traffic elements was developed by Wang et al. [40]. Traffic elements focus exclusively on vehicles, considering a maximum of the closest eight neighboring vehicles. Using both LiDAR point clouds and image data, the proposed framework was validated using three experiments that modeled different road and traffic conditions.

Gravity models were proposed by [41] to assess the complexity of the surrounding environment, where the level of driving complexity was measured as the extra cognitive burdens exerted by the traffic environment on the drivers. That said, the proposed method could not directly obtain the complexity values, and many relevant parameters were miscalibrated in the calculations. Following the same concept, Yang et al. [42] divided the environment into static and dynamic elements to develop their environment complexity model. The static features were studied using the grey relation analysis. At the same time, the complexity of the dynamic elements was quantified based on the improved gravitation model, adding an extra explanatory variable into the function to explain the degree of contribution of the driving strategy.

Focusing on the dynamic traffic elements only, ref. [43] proposed a framework that captured the objective human drivers’ judgment on the complexity of the driving environment. In this method, the complexity of the environment was defined based on the interactions of the ego vehicle with the vehicles surrounding it. Applying this framework to three case studies involving different road maneuvers showed that the produced complexity curves managed to quantify and time the changes in environmental complexity. One drawback to this method is its inability to describe the static environment complexity.

Multiple researchers utilized the potential field theory in their models. A highway potential field function was proposed by Wolf et al. [44] to aid the AV’s obstacle avoidance system. Wang et al. [45], on the other hand, proposed three different fields (moving objects, road environment, and driver characteristics). Similarly, Cheng et al. [46] based their environmental complexity evaluation model on the potential field theory. The environment elements are represented by a positive point charge or uniformly charged wires that create a potential field in their vicinity. The total potential field of a certain environment can be calculated by superimposing individual fields. The virtual electric quantity of the different environment elements is calibrated using the AHM, where non-motorized vehicles have the highest values and static traffic elements like lane markings have the lowest values. This method was verified on virtual and real traffic scenarios and showed comparable results with expert scoring.

The existing literature’s evaluation of the surrounding environment’s complexity for AVs has some limitations, such as using predefined subjective ratings for various road and weather conditions, relying on expert scorings, which is simplistic and lacks comprehensiveness, and failing to capture an accurate perception of AVs. Moreover, the available models predominantly focus on camera and video data or hypothetical scenarios, overlooking the potential of LiDAR point clouds. These models also fail to harness the fusion of multiple sensory inputs, such as LiDARs and cameras, which can be used to advance object detection for AVs [47]. Additionally, existing models calculate the complexity in arbitrary regions of interest around the AV without accounting for the specific sensor specifications.

Consequently, the proposed framework aims to offer a more insightful understanding of the static environment’s complexity. It achieves this by accurately simulating the actual perceived environment using commercially available LiDAR sensors via digital twin environments, addressing the aforementioned limitations. Furthermore, the complexity of the environment is captured via the estimation of the required processing data rates, which also anticipates the level of performance of AVs in different driving environments and weather conditions.

## 3. Methodology

**Figure 1:**
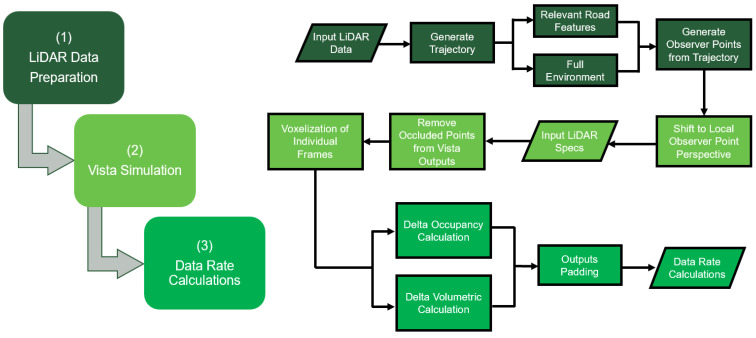
High-level framework for methodology.

The analysis will be conducted in two ways: first, it will encompass the entire scanned environment, including all road and roadside features. Second, it will focus solely on the right-of-way section of the road, containing pertinent road features. This dual approach aims to effectively gauge the influence of the road’s geometry (including vertical and horizontal curves and roadway width) on the AV’s performance. The contrasting data rate requirements between the comprehensive scene and the relevant road features will also be highlighted. An overview of the implemented methodology will be presented in this section. The general high-level framework implemented in this work is illustrated in Figure 1.

### 3.1. Point Cloud Data

The primary input of this framework is the 3D LiDAR point cloud data generated from mobile laser scanners (MLS). The data used in this research were collected by Alberta Transportation using Tetra Tech PSP-7000. This vehicle is mounted with a REIGL VMX-450 system [48] that can collect 360° LiDAR data using 2 RIEGL VMX-450 laser scanners, producing up to 1.1 million measurements/s and has up to 400 lines/s scanning rate. Finally, the scanning was conducted at normal traffic flow speeds of up to 100 kph so as not to disrupt traffic operations. The NCHRP guidelines [49] recommend a point cloud density of 30–100 pts/m^2^ and an accuracy range of 0.05–0.2 m for autonomous navigation. The density per square meter was calculated for the used road sections, and it was determined that the average density of the used data exceeded 150 pts/m^2^, surpassing the recommended limit. Furthermore, the RIEGL’s 8 mm accuracy confirms both the quality and fitness of the input data.

The used point cloud data was manually checked and verified to ascertain their integrity by comparing them to the real environment and ensuring there was no discernable loss of road information. Any excess points induced by noise are filtered to prepare the point clouds for later calculations. Finally, since this framework focuses on the interaction between the ego vehicle and the statical environment only, the influence of moving targets like vehicles, pedestrians, and cyclists was ignored. Hence, they were manually removed from the point clouds as they can influence the calculations by occluding some road points beneath and behind them.

### 3.2. Test Segments

Throughout this analysis, a wide range of terrains and road geometries provide valuable insights into the performance of AVs across diverse driving environments. The study has incorporated in its analysis sections from the following highways in Alberta, Canada, namely, AB-816 and AB-32 for vertical curve analysis, AB-3 for studying roadway width variations, and AB-11 and AB-1A for analyzing horizontal curves.

### 3.3. Trajectory Generation

**Figure 2:**
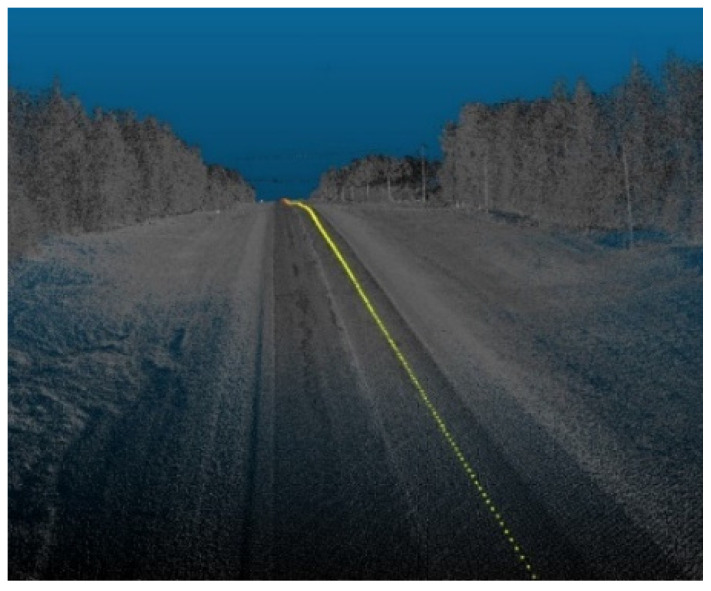
Trajectory points illustration.

The sensor must be placed in its natural position in the virtual environment, typically on top of the AV, to generate accurate simulations. In this analysis, it was assumed that the AV would follow the trajectory of the data collection vehicle by capturing the road points with a scan angle rank of zero, or in other words, the road points that lie directly beneath the vehicle that collected that point cloud data. Figure 2 shows an illustration of the generated trajectory points. 

Three direction vectors, leftward, upward, and forward vectors, were calculated for each point to obtain its directionality and orientation along the vehicle’s trajectory. Once the trajectory has been defined, the sensor can be positioned at observer points by translating any road point by a distance of 1.8 m, which takes into account the height of the ego vehicle and an additional clearance to accurately capture the position of the LiDAR sensors on top of vehicle’s roof. For detailed computations of the trajectory and direction vectors, refer to [14].

**Figure 3:**
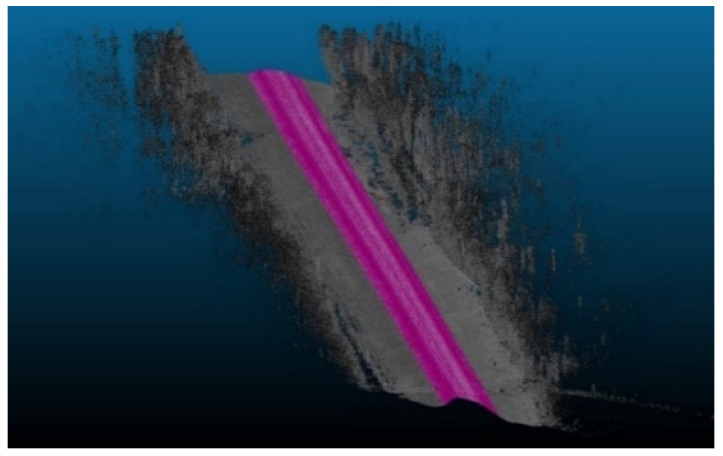
Trimmed road section (highlighted in purple).

**Figure 4:**
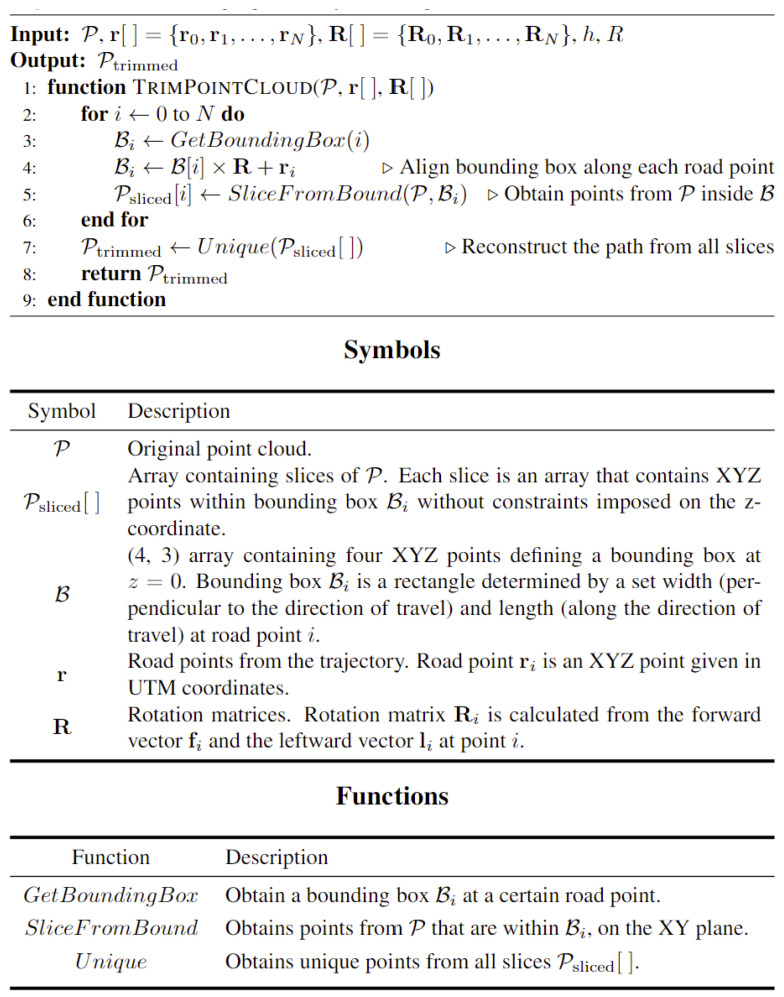
Right of way trimming.

After cleaning the road sections, the relevant road features (RRFs) section is trimmed out and extracted from the original point cloud, as shown in Figure 3. A Python code was developed to generate bounding boxes B_i_ that are centered and rotated around the trajectory points $r_{i}$, which are uniformly spaced across the point cloud P. Each bounding box B_i_ is defined with a set length and width; for the z direction, no constraints are imposed. A smooth region that simulates the right-of-way region can be extracted by aggregating the bounding boxes at each road point along the trajectory. An overview of the implemented code is outlined in Figure 4. The analysis area, based on the layout of the road, should encompass the width of the driving lanes, road shoulders, and an extra space beyond the shoulder that can extend to a couple of meters such that all road and traffic signs are present in the calculations.

### 3.4. Vista Viewpoint Synthesis

The VISTA simulator is one of many open-source, data-driven simulators available to researchers [21]. VISTA is mainly sought-after for its ability to rapidly generate novel viewpoints and simulate different types of sensors like cameras and LiDAR. VISTA offers high-fidelity, precise perspective-based viewpoints based on predefined sensor configurations, enabling the detailed visualization of the vehicle’s surroundings along its trajectory. Complete documentation of the implemented processes can be found in [50].

**Figure 5:**
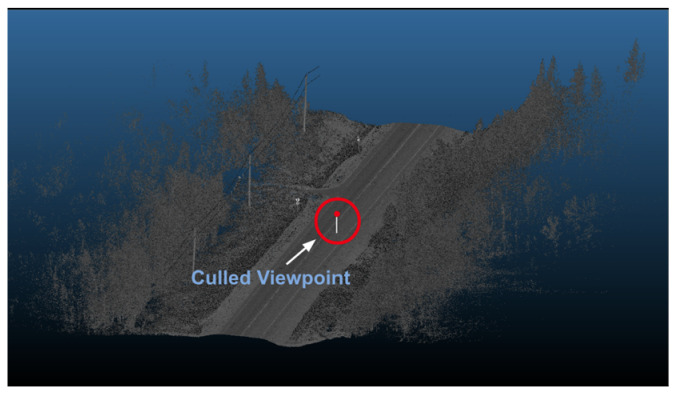
Culled points within the sensor range.

**Figure 6:**
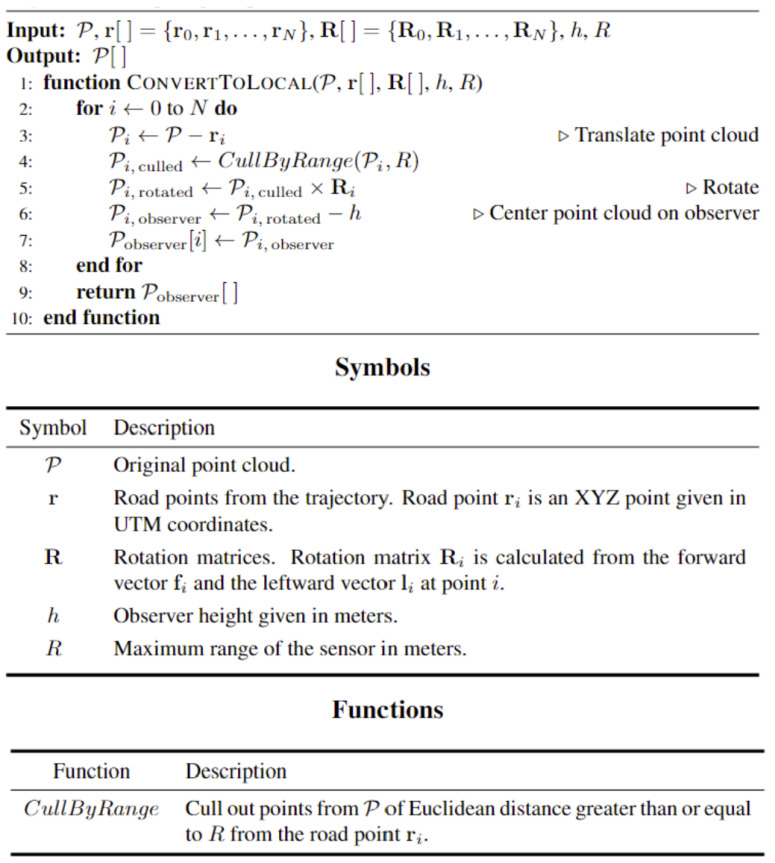
Point cloud preparation for VISTA.

First, the perspective must shift to a local road point to produce accurate synthesized viewpoints by applying a rigid transformation to the point cloud. The point cloud has to be rotated and translated using the trajectory information to implement such a transformation. Once this is complete, the Euclidean distance between the newly found origin and the road points is calculated to cull all points not within the predefined sensor range, as shown in Figure 5. Finally, the elevation of the culled road points is subtracted by 1.8 m to shift the perspective from a road point into an observer point. An overview of this process is outlined in Figure 6.

**Figure 7:**
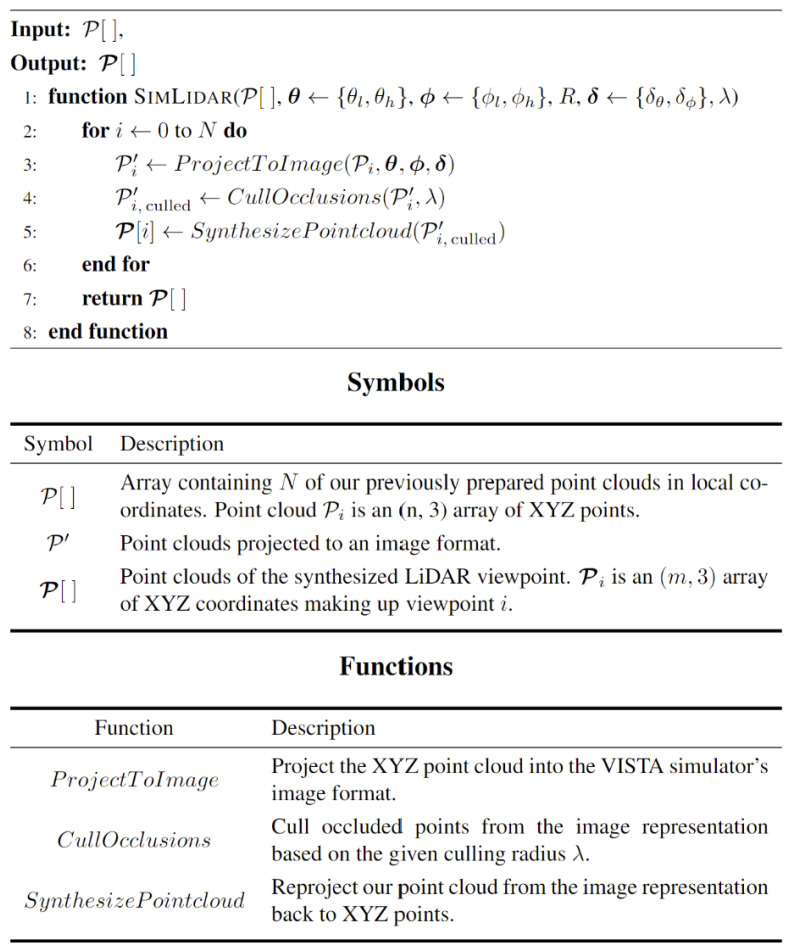
Synthesized viewpoints from VISTA.

Vista prevents foreground–background blending by transforming the 3D view into a 2D image using polar coordinates (yaw ⍺, pitch 𝛽) and calculating ray distances (d) from the observer point using Equation (1). Figure 7 summarizes the Vista viewpoint synthesis.

**Figure 8:**
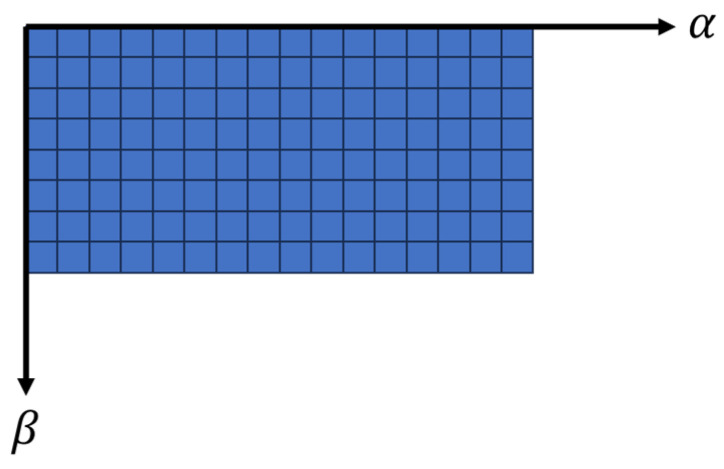
Two-dimensional image polar coordinates with yaw (⍺) and pitch (𝛽) angles.

Pixel size corresponds to sensor angular accuracy, and the total number of pixels is determined by dividing the angle range by accuracy. Consequently, higher resolution results in more pixels and fewer points per pixel. Once occlusion handling is finished, the points are reverted to the 3D view. A depiction of the 2D image representation is illustrated in Figure 8.

(1) $$⍺=\arctan(\frac{P_{i,y}}{P_{i,x}})\;\;𝛽=\arcsin(\frac{P_{i,z}}{d})\;\;d=\left|\left|P_{i}\right|\right|$$

For pixels that contain multiple points with variable depth values inside them, Vista prioritizes the point that has the least depth and assigns its value to the whole pixel. This process, in principle, is very similar to traditional raycasting techniques [37], which calculate occlusions by electing points closest to the sensor and filtering out the points in the background. However, the occlusion calculation from VISTA is more advanced as it adds an extra layer of robustness by comparing a pixel’s distance to the average distance of its neighboring arrays. If the pixel in question has a depth bigger than the adjacent pixels, it is removed as it is considered occluded.

The Culling Radius variable (λ) defines the number of neighboring pixels. Users can adjust λ, ensuring suitability for data and sensor type. λ = 0 yields standard raycasting. λ = 2 provided consistent, precise outputs within the sensor’s range, which was adopted for this study to maintain analysis accuracy.

**Figure 9:**
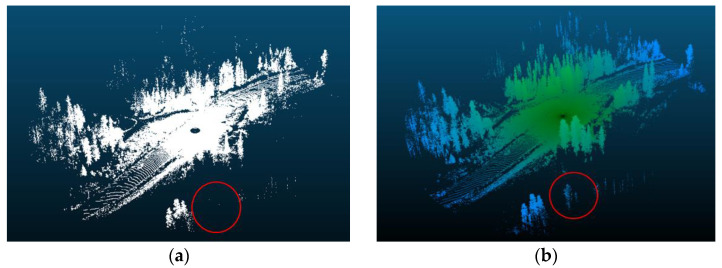
Comparison between point cloud outputs using vista and raycasting: (**a**) at λ = 2, vista accurately occludes the highlighted tree at the bottom of the frame and generally has lesser vegetation density; (**b**) raycasting output captures the majority of the highlighted tree and has much denser vegetation. Red circles highlight the location of an occluded tree.

VISTA’s superior occlusion handling outperforms traditional raycasting, as illustrated in Figure 9, which shows that VISTA accurately occludes the highlighted tree at the bottom of the frame, while raycasting fails to do so. Furthermore, raycasting displayed an unnaturally high vegetation density because raycasting allows rays to pass between obstacles, unlike VISTA. As a result, Vista was chosen as it is a more reliable tool for accurate occlusion calculations.

### 3.5. Data Rate Calculations

A recent report established that AVs must see sooner, clearer, and farther to prevent roadway fatalities. To do so, the onboard computer has to process vast amounts of real-time sensor data to accurately reconstruct the scene, enabling obstacle avoidance. The report developed an equation to estimate required data rates, capturing all the necessary elements for estimating such data rates [51]. This equation has been adopted to explain the complexity of different static driving environments, focusing on LiDAR as the primary source of sensory data and excluding the impact of dynamic objects like vehicles, cyclists, or pedestrians.

For each frame (i) in the point cloud, the corresponding data rate value can be calculated using Equation (2).

(2) $$\begin{array}{c} d\left[i\right]=\frac{R\left(\theta_{h}-\theta_{l}\right)\left(\phi_{h}-\phi_{l}\right)}{\delta_{R}\delta_{\theta }\delta_{\phi }}\cdot \frac{32Fb\Delta_{i}\log\left(\frac{1}{2\Delta_{i}}\right)}{3{\mathrm{SNR}}_{\max\;\mathrm{range}}} \end{array}$$

Since the ego vehicle’s primary source of sensory vision is the LiDAR, the considered factors are mainly related to the LiDAR equipped by the AV. In the proposed equation, (R) is the maximum sensor’s range in meters; θ is the azimuth or horizontal angle of the sensor; $\left(\phi \right)$ is the elevation or vertical angle of the sensor, and they are both in degrees; and ($\delta_{R}$, $\delta_{\theta }$, and $\delta_{\phi })$ are the range, azimuth, and elevation precisions, respectively, in meters and degrees. (F) is the refresh rate for the used sensor in hz. Analog-to-digital (ADC) LiDAR digitizes the oncoming signals to obtain range information [52], and (b) is the accuracy in bits for each sampled signal, which measures the required data rates in bits/s. For this work, a value of 12 is used. Turning to delta (Δ), it is the percentage of occupied voxels, and finally, (SNRmax) is the signal-to-noise ratio at the maximum range in dB. All the terms of the equation are sensor-specific except for delta (Δ), meaning that they can be treated as constants unless the sensor is changed because each sensor has its operational field of view dictated by its range (R).

The signal-to-noise ratio (SNR) is a term that enables the simulation of weather conditions in the analysis. It is established that LiDAR operating on higher SNR levels will enhance their detection range and precision [53]. Furthermore, it is understood that in inclement weather conditions, the performance of LiDAR is affected significantly as the medium through which the signals of the LiDAR travel tend to carry a lot of noise. Especially at longer ranges, the signal’s power reflected off a target is heavily attenuated, which might cause misdetections if the signal strength falls below the noise strength [54], thus increasing the number of computations to process the surroundings accurately and, by extension, increase the data rate requirements. For instance, as the rain intensity increases, the accuracy of the point cloud perceived by the LiDAR decreases, directly affecting the AV’s ability to detect and track objects [55].

Delta, on the other hand, is a variable that captures the density and complexity of the surrounding environment from the perspective of the equipped LiDAR sensor. It can be calculated by first voxelating the point cloud space and then finding the portion of the voxels occupied by points. A voxel is either occupied or empty, so if a single voxel is occupied by more than one point, only a single point is kept, and the rest of the points are removed from the calculations.

The density of the points at any region is dictated by angular precision, which, in principle, controls the minimum gap between two consecutive distinguishable points in the cloud. Naturally, the spacing between two neighboring laser beams increases away from the sensor since they are all fired from the same location. Hence, this gap reaches its greatest value at the maximum sensor range, leading to a notable reduction in point density. Conversely, the gap is minimal near the sensor, resulting in significantly higher point cloud densities [56].

**Figure 10:**
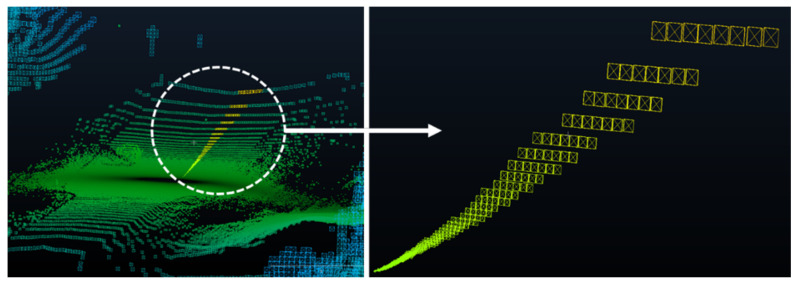
Varying voxel sizes along the sensor’s range.

**Figure 11:**
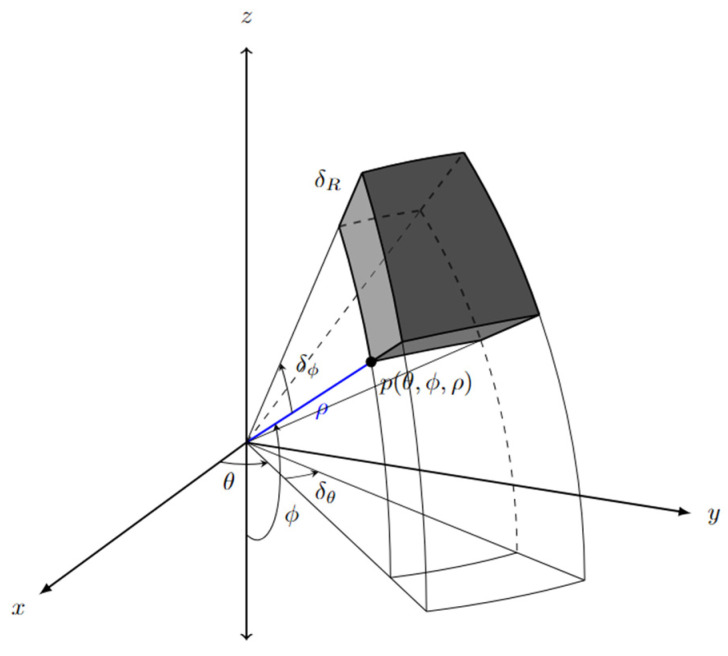
Voxels in spherical coordinates.

Voxelization was carried out in spherical coordinates to simulate the ring pattern of LiDAR and the inherent sparse nature of points that are distant from the sensor [57]. The efficient accommodation of varying point density minimizes information loss and maintains accuracy in the analysis [58]. This was achieved by changing the voxel size along the sensor’s range depending on its position, as shown in Figure 10. The voxel size is contingent on the LiDAR sensor’s precision in the simulations. As demonstrated in Figure 11, each point in space is defined by azimuth (θ) and elevation (∅) angles, along with the range (ρ), which is the Euclidean distance from the observer point. Converting the cartesian system to the spherical system can be carried out using Equation (3).

(3) $$\begin{array}{c} P\left(x,y,z\right)\Rightarrow P\left(\rho ,\theta ,\phi \right)\left\{\begin{array}{c} \theta =\tan^{-1}\left(\frac{y}{x}\right)\\ \phi =\cos^{-1}\left(\frac{z}{\sqrt{x^{2}+y^{2}}}\right)\\ \rho =\sqrt{x^{2}+y^{2}+z^{2}} \end{array}\right. \end{array}$$

**Figure 12:**
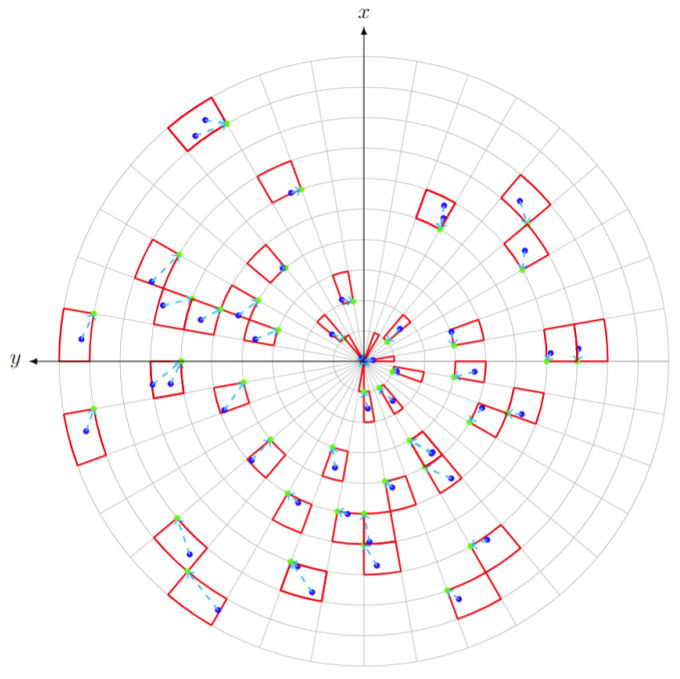
Planar view for occupied voxels and their base points: the points captured by the sensor are in blue, the occupied voxels are in red, the green points correspond to the unique base point of the voxels, and the cyan dotted lines refer to the respective voxel index.

Identifying the locations of all occupied voxels in the space involves calculating the indices of each occupied voxel. Then, the floor of the indices is taken since they correspond to specific locations on the spherical grid; hence, they have to be integers. In doing so, the points are grouped into their respective voxels. The voxel index corresponds to the location of the occupied voxel in the spherical grid and can be obtained by dividing the spherical coordinates of each point by their respective precisions ($\delta_{R},\;\delta_{\theta },\;\delta_{\phi })$ to obtain their projection inside the spherical grid system defined by the sensor configuration. Figure 12 provides an overview of the depiction of occupied voxels in the spherical coordinate space.

The primary methodology employed for this analysis centers around the occupancy method, where points are assigned equal weights of contribution regardless of their position from the sensor. However, the proximity of points to the sensor results in a higher contribution due to their dense presence. Conversely, the contribution of more distant points is lower, attributable to their sparser distribution. In this method, the calculation of the delta involves determining the ratio of the total number of occupied voxels (k) to the total number of voxels within the space. The delta value for each frame (i), following the occupancy approach, can be calculated using Equation (4).

(4) $$\Delta_{i}=\frac{k}{\left\lfloor \frac{\theta_{h}-\theta_{l}}{\delta_{\theta }}\rfloor \times \lfloor \frac{\phi_{h}-\phi_{l}}{\delta_{\phi }}\rfloor \times \lfloor \frac{R}{\delta_{R}}\right\rfloor }$$

The volumetric method, however, adopts a different approach in calculating delta where more weight is assigned to the distant points rather than the closer ones to account for the scarcity in their numbers. This calculation is performed by utilizing the voxel size, which is maximum at the sensor’s maximum range and minimum in the vicinity of the sensor. Hence, the contribution of the distant points will be high owing to their bigger size. The distant points pose a challenge for AVs because their accurate detection is inherently difficult [59].

The corresponding delta can be computed by dividing the aggregate volume of all occupied voxels ${𝒱}_{𝒾}$ by the total volume (*V*) defined within the bounds of the sensor’s field of view (FOV). To obtain ${𝒱}_{𝒾}$, the first step involves reverting the voxel indices to spherical coordinates by multiplying the voxel indices by their respective precisions, which results in the base points $p_{i}$ of all occupied voxels where $p_{i}=\left\{p_{1},p_{2},\ldots ,p_{k}\right\}\left(\theta ,\phi ,\rho \right)$. From the voxel representation $v_{i}$, for each unique voxel’s base point (j), the volume of the occupied voxel can be calculated via integration by knowing the precisions of the sensor. Upon calculating the volume of all occupied voxels up the kth base point, ${𝒱}_{𝒾}$ can then be obtained by summing the volume of the occupied voxels as seen in Equation (5):

(5) $${𝒱}_{𝒾}=\overset{k}{\underset{j=1}{\sum }}\int_{\rho_{j}}^{\rho_{j}+\delta_{\rho }}\int_{\phi_{j}}^{\phi_{j}+\delta_{\phi }}\int_{\theta_{j}}^{\theta_{j}+\delta_{\theta }}\rho_{j}^{2}\sin\phi_{j}\;d\theta \;d\phi \;d\rho$$

Finally, delta can be calculated using the volumetric approach by dividing the volume of occupied voxels by the total volume covered by the sensor FOV using Equation (6).

(6) $$\Delta_{i}=\frac{{𝒱}_{𝒾}}{\int_{R_{l}}^{R_{h}}\int_{\theta_{l}}^{\theta_{h}}\int_{\phi_{l}}^{\phi_{h}}R^{2}\sin\phi \;d\phi \;d\theta \;dR}$$

### 3.6. Padding Region

Owing to the 360° vision of the LiDAR sensor, the surroundings can be effectively scanned both ahead and behind the sensor, with a distance equivalent to its operational range. Hence, to overcome the problem of having a start and an end to the test segments, where at the beginning of the road section, the region behind the sensor is typically empty, and conversely, at the end of the road section, the region ahead of the senor is also vacant. A solution is devised via the introduction of padding regions at these extremities of the test segments. These regions are introduced at both the start and the end of the road section and have a range equivalent to the sensor’s range. In doing so, the consistency of the calculations will not be compromised, as this will ensure that the calculation region for each frame along the road section is constant and equal to twice the sensor’s range.

### 3.7. Sensor Specifications

**Figure 13:**
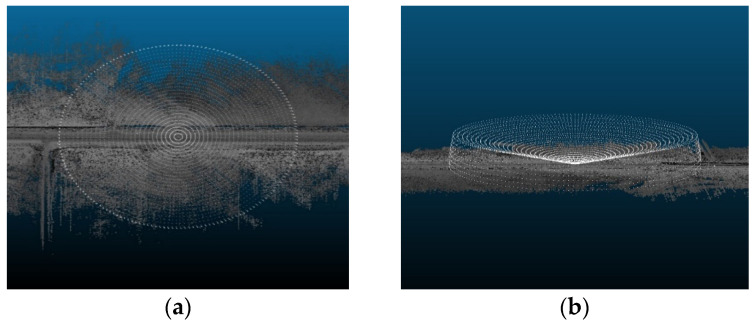
VLS-128 LiDAR sensor FOVs: (**a**) top view; (**b**) side view.

The various parameters of the sensor specifications used in the simulations were modeled on real-world sensors. In particular, the state-of-the-art Velodyne Alpha Prime LiDAR [60] sensor, also known as VLS-128, is used as the primary sensor for this framework, whereas HDL-32E will be used for comparison. Table 1 illustrates the adopted sensor specifications. Figure 13 demonstrates a graphical representation of the VLS-128 model FOV.

## 4. Results

The simulations implemented in this analysis are divided into two folds. The first fold comprises the full environment (FE) analysis, and the second fold focuses on the road’s main relevant features (RRFs). This approach enables a comparative evaluation of results, elucidating the extent of additional computational demands arising from extraneous roadside elements like vegetation. The outputs of this analysis measure the expected values for the data rate requirements at each frame of the road section. The analyzed frames are spaced 1 m apart across the road section. Furthermore, images from Google Maps Street View [61] will be provided along with the graphical data rate requirements outputs to validate that the analysis findings are reflected in the real-world environment.

### 4.1. Relevant Road Features

#### 4.1.1. Vertical Curves

**Figure 14:**
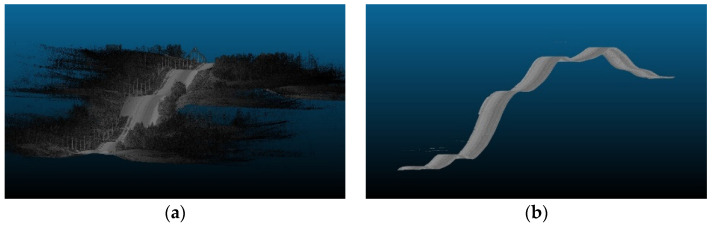
Example of vertical curves analysis sections: (**a**) full environment (FE) of road section (1); (**b**) relevant road features (RRFs) of road section (1).

The primary objective of this analysis is to examine the impact of vertical curves on the data rate requirements. Vertical curves, standard features found in most roadways, present a challenge for autonomous vehicles (AVs) because of their potential to compromise the line of sight or reduce visibility due to occlusions. Understanding the implications of these curves is crucial for ensuring AVs’ safe and efficient operation. Figure 14 showcases the general layout of one of the analyzed road sections. This analysis aims to investigate the influence of the vertical curves on the data rate requirements and overall performance of AVs in a controlled environment.

**Figure 15:**
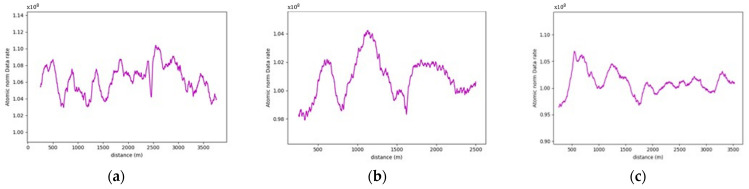
Data rate requirements graphs for vertical curve analysis sections: (**a**) road section (1); (**b**) road section (2); (**c**) road section (3).

**Figure 16:**
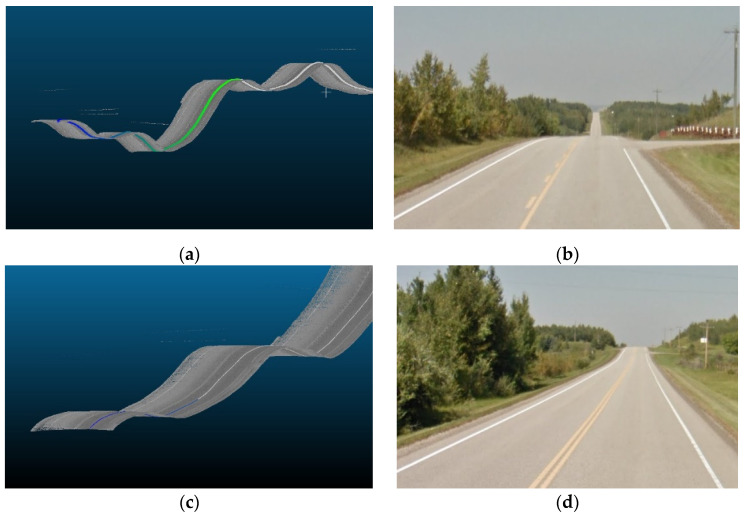
Demonstration of critical locations in road section (1): (**a**) frame 1525 (crest) location; (**b**) frame 1525 on Google Maps; (**c**) frame 480 (sag) location; (**d**) frame 480 on Google Maps.

On studying the data rate requirements graph for the road sections, a pattern was observed where the data rate values were, on average, lower at the crest vertical curves when compared to the sag vertical curves due to occlusion from the road geometry, as seen in Figure 15. For instance, as illustrated in Figure 16, the lowest values in the first road section were traced to different crest locations, such as frames 665 and 1525. Conversely, the highest values on the graph, for example, at frames 480 and 881, were located on sag curves. Similar trends were evident in the second road section. Once again, on average, the data rate values at sag curve locations exceeded those at crest curves. Frames 824 and 1621, located on crest curves, recorded the lowest values, whereas frames 600 and 1135 reported the highest values and were situated on sag curves. The third road section was no exception, and the same pattern was observed where frames falling on sag curves, such as 543 and 1240, had higher values than frames 257 and 1700, which were situated on crest curves.

**Figure 17:**
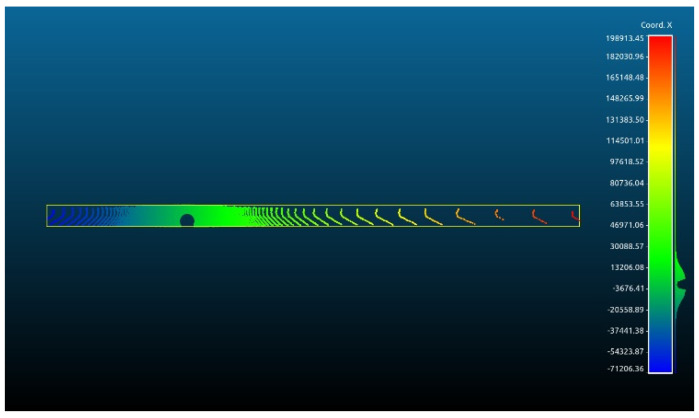
Road section (1)—Frame 1525 plan view Vista outputs (vision range in mm): the field of vision extends from approximately 72 m behind the sensor to 198 m in front of the sensor.

Naturally, the visible range of the sensor is controlled by the geometry of the road, where the grade percentage is a key feature. For instance, the crest curve at frame 1525 in the first road section was characterized by +3.6% and −0.8% tangents. As illustrated in the vista outputs in Figure 17, the range of vision on the −0.8% slope extended to approximately 200 m, while the range on the +3.6% slope was restricted to 72 m only.

By investigating the number of points captured within the sensor’s short range and comparing them to the total number of points within an individual frame, it was established that, as a general trend, when only the RRFs are considered, approximately 95–97% of the captured points are located within the initial 50–60 m around the sensor.

**Figure 18:**
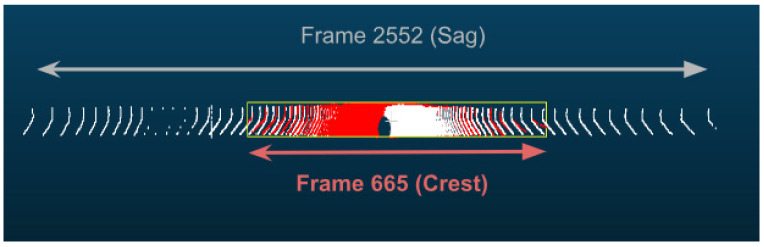
Road section (1)—comparing the extent of vision between frame 2552, located on a sag curve (depicted in white), and frame 665, located on a crest curve (depicted in red).

This observation is exemplified by comparing frames 2552 and 665 in the first road section, situated on sag and crest curves, respectively. As shown in Figure 18, the vista outputs of frame 2552 (depicted in white) revealed a range of vision extending to 200 m on each side, whereas it was constrained to 93 m in frame 665 (shown in red). Frame 2552 contained 44,500 points, while frame 665 had 41,300 points. The number of points beyond the 90 m range of vision in frame 665 amounted to only 756 points. This quantity constitutes a mere 1.6% of the total points in frame 2552. This quantity constitutes a mere 1.6% of the total points in frame 2552. Such observations align with the typical behavior of LiDAR sensors, wherein the number of points in the far range is significantly lower than those close to the sensor, thus explaining why there are small margins among the data rate values in the vertical curve analysis, especially when considering the RRFs only.

Table 2 summarizes the comparison between the values at the different vertical curve types to the values at the flat section of the road, where a maximum of 7% increase in values is recorded at sag curves, as for crest curves, a maximum of 4.2% decrease in values was recorded.

#### 4.1.2. Roadway Width

**Figure 19:**
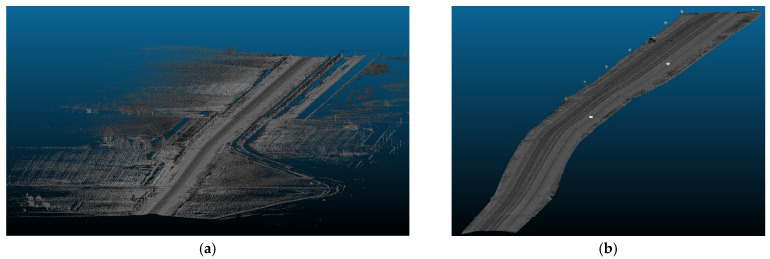
Example of roadway width analysis sections: (**a**) full environment (FE) of road section (4); (**b**) relevant road features (RRFs) of road section (4).

**Figure 20:**
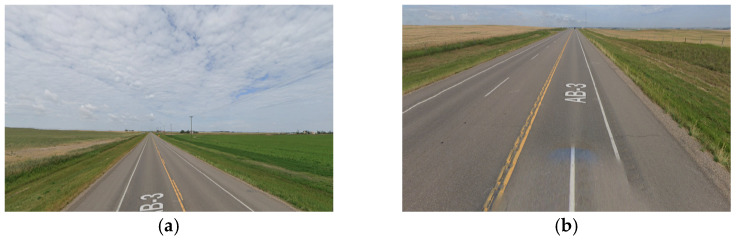
Illustration of two-way, two-lane highway: (**a**) typical 2-lane zone; (**b**) passing lane zone.

The primary research question for this study centers on how alterations in roadway width affect the data rate requirements, especially in areas where the number of lanes increases or decreases. Within this study, four distinct road sections, each having a length of 4 km, situated on the same highway were examined. Figure 19 provided a visualization of one of the analyzed road sections. As seen in Figure 20, the selected road sections are mainly configured as two-way, two-lane roads with passing lanes. The average data rate requirements in the 2-lane zones will be compared to those in the 3-lane zones to quantify the extent of change in the data rate requirements between the two distinct zones.

**Figure 21:**
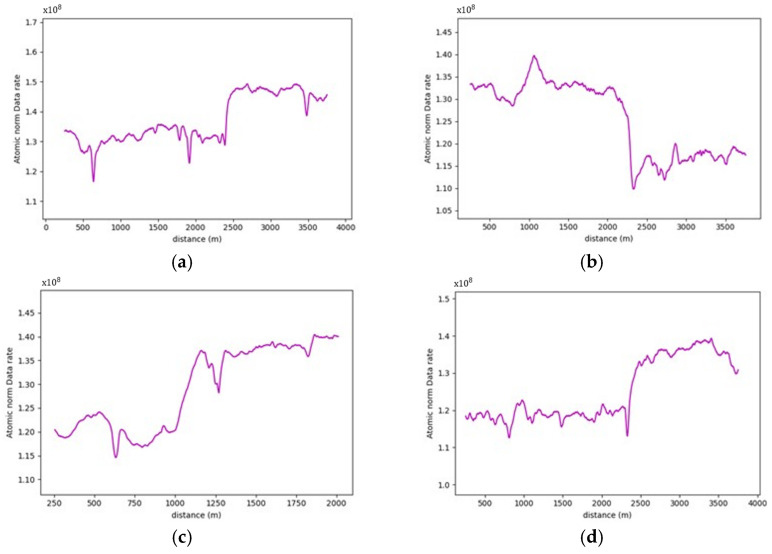
Data rate requirements for road sections experiencing variation in roadway width: (**a**) road section (4); (**b**) road section (5); (**c**) road section (6); (**d**) road section (7).

More often than not, the general layout of the road geometry changes along any trip. This change is vividly depicted in the data rate values seen in Figure 21. A pattern has been established where the average data rate requirements at the two-lane zones are lower than those at the three-lane zones.

For instance, road section (4) began with a standard two-lane configuration, and approximately halfway through this section, a passing lane was introduced in one direction. This change is reflected on the data rate value graph, which clearly illustrates an increase in data rate values as the AV enters the three-lane zone. In the two-lane zone, data rate values averaged $1.30\times 10^{8}$ bits/s. In contrast, within the three-lane zone, data rate values averaged roughly $1.46\times 10^{8}$ bits/s. This difference indicates an average increase in data rate values of approximately 12.3%. The summary of the data rate values for the analyzed sections is outlined in Table 3, which shows that adding an extra lane would require the AV to process an extra 12.3–16.5% additional data to maintain its safe operations, making the environment more complex.

**Figure 22:**
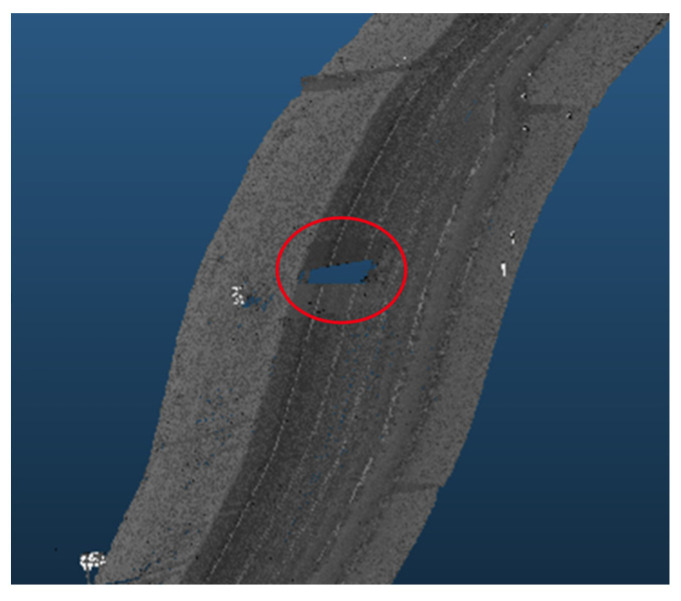
Example of roadway width analysis sections: the red circle demonstrates a gap in the road pavement.

It is important to note that the displayed graphs experienced some abrupt drops in data rate values, which were associated with temporary loss of road information due to obstructing vehicles leaving gaps on the road during data collection, as shown in Figure 22. These localized drops, however, were not factored into the calculations.

### 4.2. Full Environment

#### 4.2.1. Horizontal Curves

The following analysis delves into horizontal curves and their influence on AVs’ data rate requirements. The analysis encompasses the complete surrounding environment around AVs. Unlike controlled environments, real-world scenarios often present challenges like buildings, dense vegetation, and towering mountains. These features obstruct sightlines, rendering horizontal curves particularly critical for human drivers and AVs because AVs rely heavily on sensor data and real-time processing to navigate these curves safely. Therefore, it is necessary to account for such obstructions to derive accurate conclusions regarding the impact of horizontal curves on data rate requirements. Typically, when the sight distance is compromised, AVs experience a loss of crucial information regarding potential obstacles on the road.

**Figure 23:**
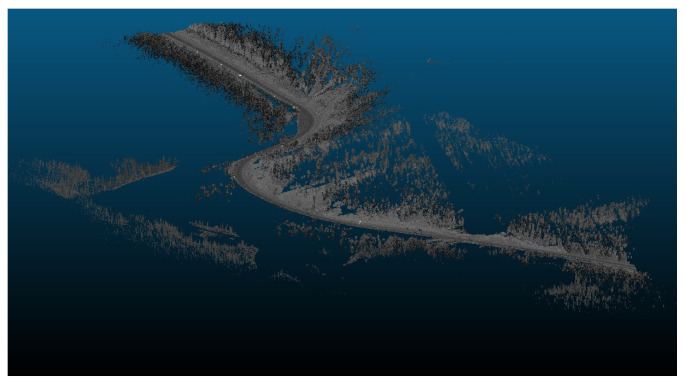
Full environment of road section (8) for horizontal curve analysis.

Three road sections comprising critical horizontal curves and situated on a mountainous terrain were considered for this analysis. The visualization for one of the analyzed road sections is depicted in Figure 23. Although the presence of the vegetation and other offroad elements in the analysis are expected to have random variations on the data rate values as their density is inconsistent and unevenly distributed along the road, the effect of the critical horizontal curves was still observed, primarily due to the anticipated occlusions at these locations.

The average data rate values from the areas close to the critical curve were calculated and compared to those at the horizontal curve locations as they would have a comparable layout. The analysis focused on studying the impairment of both the forward and backward sensor vision at the entrance and exit of curves respectively. The observed variations before and after the curves emphasized the impact of occlusions caused by horizontal curves, resulting in a notable decreases in data rates. This approach attempts to isolate the effect of the horizontal curves by ensuring that there is no significant change in the road layout between the comparison locations.

**Figure 24:**
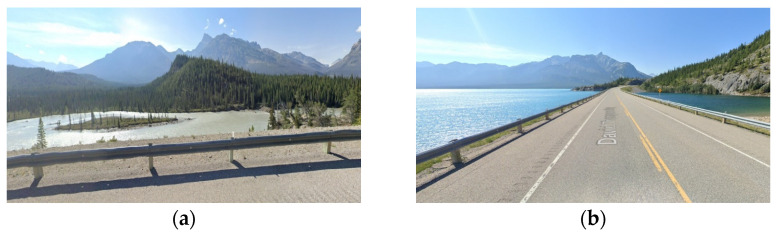
Water bodies surrounding road sections causing gaps in the point clouds: (**a**) road section (8); (**b**) road section (9).

**Figure 25:**
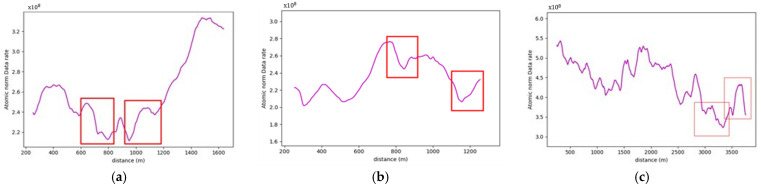
Data rate requirements graphs for horizontal curve analysis sections: (**a**) road section (8); (**b**) road section (9); (**c**) road section (10). Colored boxes refer to the entrance and exit locations, respectively, for the horizontal curves.

The presence of water bodies in road sections (8,9), as demonstrated in Figure 24, contributed to lowering their data rate requirements when compared to road section (10), which was fully covered with roadside elements. More importantly, as illustrated in Figure 25, the data rate values near the entrance of the horizontal curves decreased as the occlusion started to affect the forward vision of the vehicle by hiding some road information from the sights of the vehicle. Similarly, at the exit of the curve, the data rate values were seen to increase again because, as the vehicle moves away from the curve, the influence of occlusion on the backward vision of the sensor starts to dissipate, and the vehicle starts regaining its operating vision capacity.

This pattern was established in the three analyzed road sections. For instance, in road section (8) at frame 795, it was observed that only 120 m of the road ahead was visible when compared to the 180 m of the backward vision. This disparity was responsible for a drop in the data rate values by 16% as the values went from $2.48\times 10^{8}$ bits/s to $2.13\times 10^{8}$ bits/s. Once the vehicle was inside the curve, the vehicle could capture more of the roadside elements, which in this case are mountains on both sides of the road, which is why a slight increase in values was observed. At the exit of the curve around frame 952, the backward vision of the sensor was limited to around 110 m, while the forward vision of the road was around 175 m. From the graphs, the data rate values just after the curve, where the general layout of the environment was similar to that at the curve, were $2.43\times 10^{8}$ bits/s while the value at the exit of the curve was $2.11\times 10^{8}$ bits/s, which means there is about a 15% drop in data rate values at this position.

**Figure 26:**
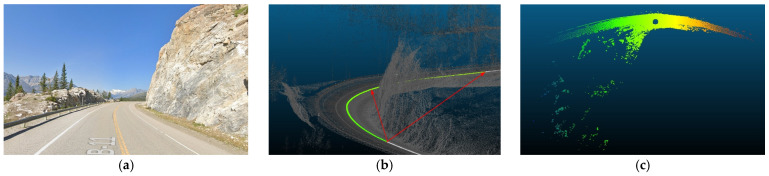
Road section (8) horizontal curve entrance at frame 795: (**a**) forward vision from Google Maps; (**b**) forward vision from the point cloud (red arrows indicate the max vision range); (**c**) Vista output for frame 795.

**Figure 27:**
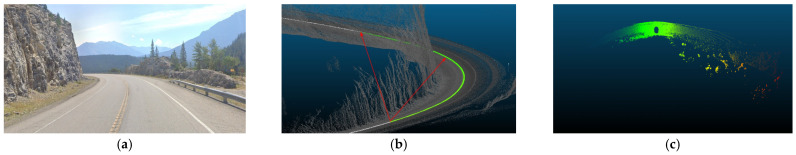
Road section (8) horizontal curve exit at frame 925: (**a**) backward vision from Google Maps; (**b**) backward vision from the point cloud (red arrows indicate the max vision range); (**c**) Vista output for frame 795.

The established pattern was also visible for the other two road sections. It is worth noting that section (10) comprised two horizontal curves: one at the start of the road section, where no roadside obstruction was recorded, and the other near the end. The second horizontal curve was identified as critical due to roadside sight obstructions. Inside the curve, the roadside element density increased as the vegetation turned into a rocky slope, so an increase in the data rate values at the center of the curve was observed. At the end of the road section, a water body replaced the vegetation on one side, resulting in a sudden drop in the data rate near the section’s edge. The average loss of information for the three-road section, as listed in Table 4, was 12.2–15.6% at the entrance of the curve and 12.7–19% at the exit (Figure 26 and Figure 27).

#### 4.2.2. Influence of Roadside Features

This analysis will compare the full environment (FE) data rate requirements and the relevant road features analysis (RRFS). Two distinct observations became apparent in this comparison. Firstly, the influence of vertical curves, whether sag or crest curves, and roadway width were generally masked, and the previously established patterns were hardly visible. Secondly, data rate requirements increase substantially if the analysis considers the full scene.

**Figure 28:**
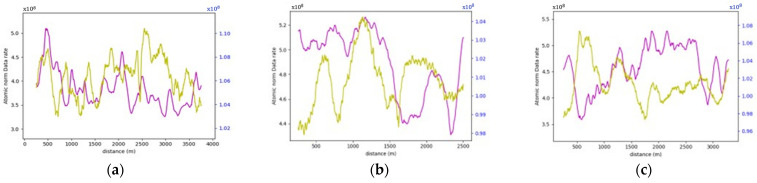
Data rate requirements for full environment analysis (purple + left axis) vs. relevant road features (yellow + right axis): (**a**) road section (1); (**b**) road section (2); (**c**) road section (3).

**Figure 29:**
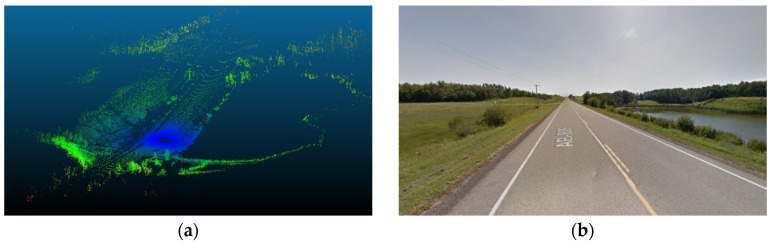
Full environment illustration of frame 880 in road section (1): (**a**) Vista output (8); (**b**) forward vision on Google Maps.

**Figure 30:**
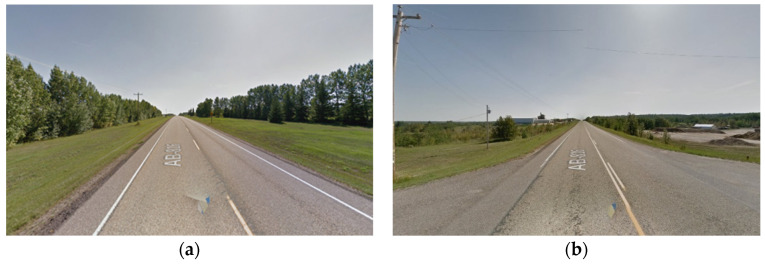
Full environment forward vision illustration examples for road section (3): (**a**) high vegetation density at frame 257 (8); (**b**) low vegetation density at frame 543.

As illustrated in Figure 28, upon examining the vertical curves of road section (1), frame 880 was situated on a sag curve, resulting in a high value in the RRF analysis. However, in the FE analysis, the density of surrounding vegetation was low at this location, and the right side of the road was occupied by a waterbody, as demonstrated in Figure 29. This configuration led to low data rate values. Similarly, in road section (3), frames 257 and 543 serve as prime examples to illustrate the limited impact of road geometry in the presence of roadside features. Frame 257, positioned on a crest curve, yielded lower data rate values than frame 543, situated on a sag curve in the RRF analysis. Nevertheless, this pattern was reversed in the FE analysis since the vegetation density at frame 543 was the lowest across the entire road section, which resulted in recording the lowest data rate requirements. Figure 30 shows the difference in the vegetation density between the two frames.

**Figure 31:**
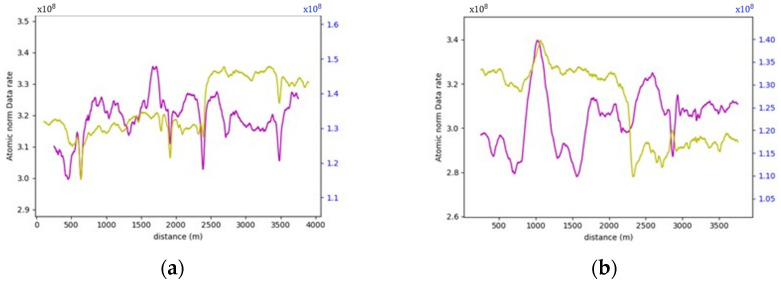
Data rate requirements for FE analysis (purple + left axis) vs. RRFs (yellow + right axis): (**a**) road section (4); (**b**) road section (5).

**Figure 32:**
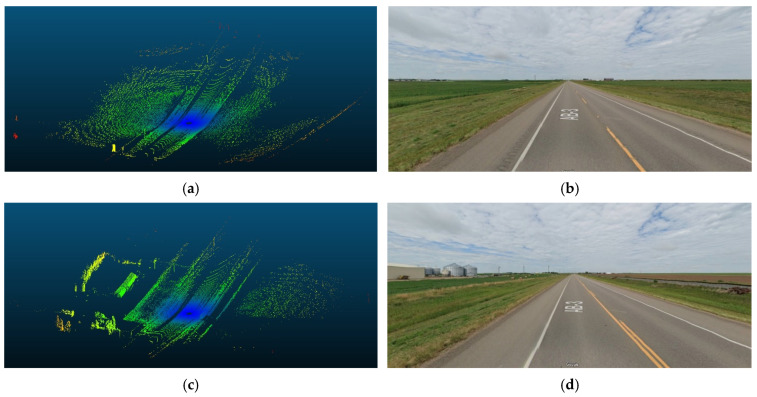
Lowest and highest frames in the full environment analysis of road section (4): (**a**) Vista output of frame 500 (lowest value); (**b**) forward vision on Google Maps for frame 500; (**c**) Vista output of frame 1700 (highest value); (**d**) forward vision on Google Maps for frame 1700.

Similarly, in the roadway width analysis, the previously observed patterns, when the number of lanes increased, was masked by the addition of the roadside elements in the analysis, as witnessed in Figure 31, which compares the data rate requirements with and without the irrelevant roadside elements. It was also noticed that the roadside features heavily influenced the data rate values. For example, in road section (4), the density of the point cloud was highest around the 1.7 km mark. Therefore, the data rate requirements were maximum, albeit in the 2-lane zone. On the other hand, around the 0.5 km mark, the low density of the point cloud prompted the data rate values to plummet. Figure 32 demonstrates the difference in the environment in these designated regions. Finally, the same inferences can be drawn from the analysis of the other road sections, where it was readily apparent that the established patterns in the variation in the roadway width were virtually nonexistent.

On comparing the data rate requirements to quantify the influence of roadside features on the performance of the AVs in rural areas, especially in scenarios where WVCs are to be avoided, the extent of additional computational burdens varied based on the surrounding environment where regions with dense vegetation would exert more computational requirements. As demonstrated in Table 5, analyzing the entire scene instead of focusing solely on relevant features is anticipated to at least double the data rate requirements. Road sections with consistently low vegetation density, such as sections (4–7) or (8–9) due to the presence of waterbodies, impose minimal extra computational burdens. Conversely, areas with high and consistent vegetation density along the vehicle’s trajectory, exemplified by frames (1–3 and 10), exhibit a significant increase in the complexity of the surrounding environment. These regions anticipate an increase in data rates up to fivefold. This study underscores the inherent challenges in handling WVCs, emphasizing the formidable computational power required for safe and efficient operations in environments marked by intricate features.

### 4.3. Sensitivity Analysis

#### 4.3.1. Weather Conditions

Favorable weather conditions are maintained throughout the analysis. In this section, an additional heavy rain environment will be modeled to showcase how much of an impact it would have on the calculations. The relationship between the ${\mathrm{SNR}}_{\max\;\mathrm{range}}$ (SNR) parameter and the data rate requirements are inversely proportional, as outlined in Equation (2). Hence, for instance, a 10% increase in the ${\mathrm{SNR}}_{\max\;\mathrm{range}}$ value will be reflected as a similar 10% decrease in the data rate requirements.

**Figure 33:**
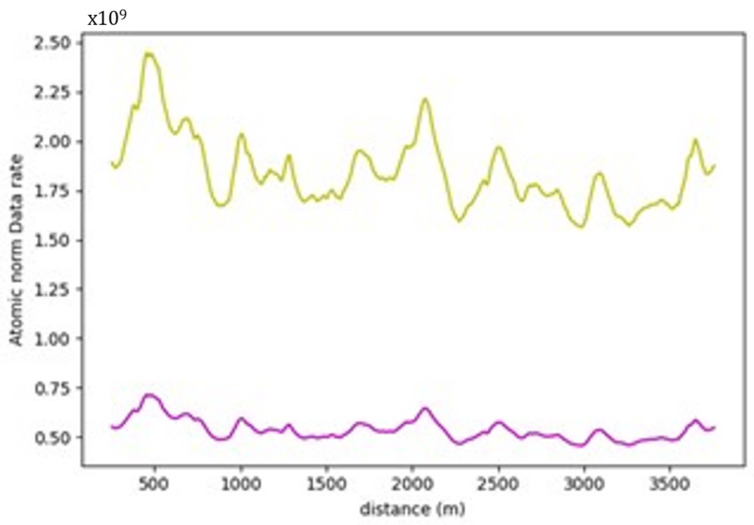
Road section (1) FE analysis. SNR = 3.5 dB (yellow curve) vs. SNR = 12 dB (purple curve).

Following the observations of [62] and knowing that the max range of the used sensor is 245 m, the SNR is expected to decrease by approximately 8.5 dB. This result would mean that the new SNR will be 12 − 8.5 = 3.5 dB. Figure 33 shows the data rate values for the same section on the same graph. Through the analysis of road section (1) with the full environment, it can be noticed that using an SNR value of 3.5 dB to simulate the effects of heavy rain, the data rate values have increased significantly by approximately 3.4 times (240%). Worse weather conditions can impose situations where the signal power might be less than the ambient noise. For instance, if the noise level exceeds the signal strength by tenfold, the SNR will be 0.1, indicating a much-weakened signal. Consequently, this condition will lead to a substantial surge of 120 times in data rate requirements, according to Equation (2). Such heightened data rates can significantly destabilize the performance of autonomous vehicles, as processing such extensive data volumes in real time will be infeasible, compromising their operational stability.

#### 4.3.2. Alternative LiDAR Sensor

To demonstrate how variations in the LiDAR sensor specifications can impact data rate calculations and affect the performance of AVs, the Velodyne-32E LiDAR will be considered an illustrative example [63]. The specifications for this sensor are detailed in Table 1. Notably, this sensor, also known as HDL-32E has a shorter range, limited to 100 m, a distinct vertical field of view (FOV) distribution, and a reduced vertical resolution of 1.33° instead of 0.11°. However, it has a slightly better range precision and matches the horizontal angle precision of the VLS-128.

The reduced vertical angular resolution of the HDL-32E LiDAR will significantly decrease the number of data points it can capture. This limitation arises because the gap between consecutive laser beams widens as the vertical precision increases. The shorter range also reduces the total number of captured data points. Consequently, the scenes captured by this sensor will, generally, be less complex for the onboard computer to process since they contain much less information than the VLS-128 model. This outcome was evident when comparing the data rate requirements for both sensors for the same road sections. In road section (2), for example, the maximum drop in the data rate requirements has been quantified as approximately 620%. This dramatic drop in data rate values means that the scenes captured by the HDL-32E LiDAR will be much less complex than those of the VLS-128 sensor.

For comparison, the analysis will keep the default padding region of 245 m instead of using 100 m to focus on the same areas of interest as those considered in the VLS-128 model calculations, facilitating a direct comparison between the two LiDAR sensors.

**Figure 34:**
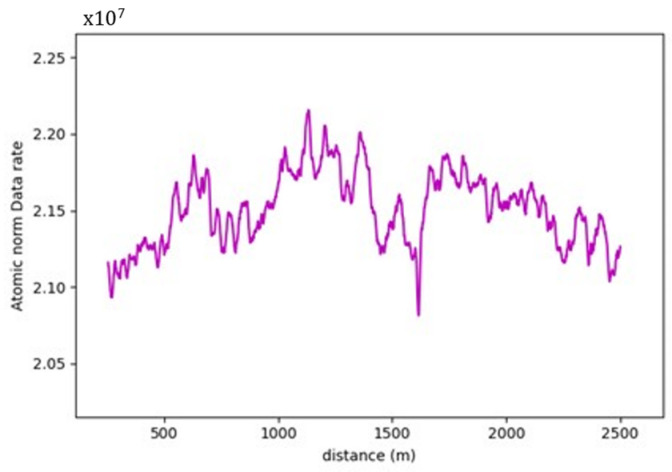
HDL-32E RRF data rate requirements for road section (2).

**Figure 35:**
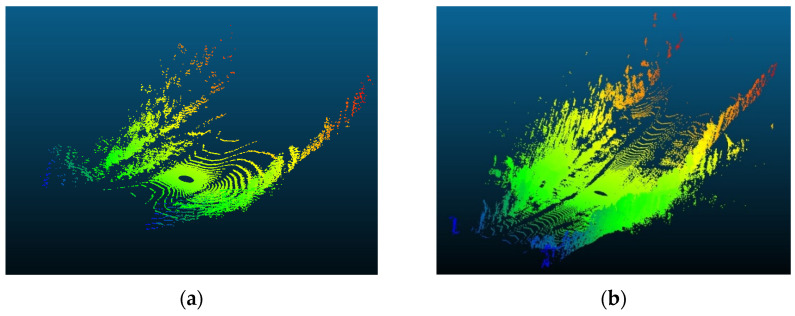
Comparison between the FE environment vista output for frame 1135 in road section (2): (**a**) HDL-32E results; (**b**) VLS-128 results.

Furthermore, the established vertical curve pattern was missing from the results, as shown in Figure 34, due to the short range of the sensor, where it did not seem to be impacted by occlusion from the road geometry. In addition, the number of captured points in the far range of the sensor is expected to be significantly lesser than those captured in the long range by the VLS-128 sensor. Hence, there will be even less impact on the occluded part of the road. To highlight the disparity in scene perception by the autonomous vehicle (AV) using various sensors, the Vista outputs for frame 1135 are presented in Figure 35. This particular frame was selected because it yielded the highest data rate value when using the VLS-128 sensor, serving as a prime illustration of the contrast between the sensor outputs. The VLS-128 sensor, undoubtedly, carries much more information for the AV and will exert more considerable burdens on the onboard computer.

**Figure 36:**
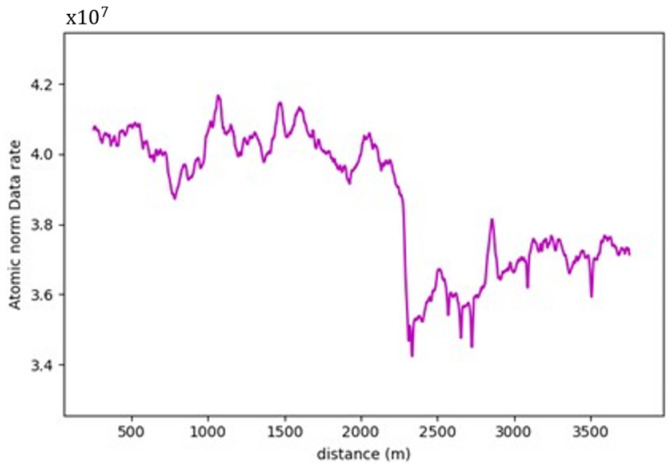
HDL-32E RRF data rate requirements for road section (5).

As for the roadway width analysis, where there are no discernible occlusion effects, it was found that the same established pattern between the 2-lane and 3-lane zones is manifested, as shown in Figure 36, where a 12.9% increase in data rate values was observed. This increase, in turn, shows that even for weaker sensors, the onboard computer is bound to experience a surge in data rate requirements when the roadway width increases.

**Figure 37:**
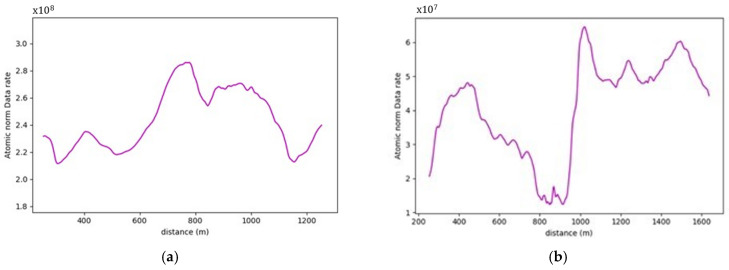
Data rate requirements for road section (9): (**a**) VLS-128 outputs; (**b**) HDL-32E outputs.

The alternative sensor did not encounter any horizontal curve occlusions for the horizontal curve analysis, as shown in Figure 37. As a result, it did not experience a reduction in data rate requirements upon entering the horizontal curve (at frame 844). In contrast, this location saw a discernible increase in data rate requirements. Whereas upon exiting the curve, an increase in values was naturally expected. However, this elevation was primarily attributed to changes in roadside vegetation density, showing that while the surrounding roadside features, undeniably, influence changes in data rate requirements, horizontal curve occlusion also plays a significant role in shaping data rate behavior.

Finally, using an inferior sensor will generate lower quality and less dense output, meaning that the AV might not be able to fully comprehend its environment from the scarce information it receives from the sensor. This, in turn, can hinder its environment perception capabilities and increase the safety risks.

## 5. Discussion

Within the comprehensive analysis of this research, encompassing diverse road sections set in multiple environmental contexts and varying sensor specifications, a central conclusion emerges: reporting absolute data rate requirements for static environments is exceedingly challenging. This complexity arises from many influential factors, including the quality and density of the point cloud data employed in simulations. The denser the point cloud is, the higher the reported data rate requirements. Additionally, the specifications of the utilized sensors, variables related to road terrain and occlusions, and the prevailing weather conditions all add to the complexity. It is also worth noting that the chosen calculation methodology, whether based on occupancy or volumetric considerations, can significantly impact the resulting data rate requirements.

On the subject of the determined data rate values, the correlation between data rate values and environmental complexity is not straightforward. Higher data rates do not always mean a more complex environment. For example, locations like horizontal curves may have lower data rate requirements, but they pose challenges for autonomous vehicles. Simply looking at decreased data rate requirements does not fully capture complexity. Instead, evaluating the percentage of missing information is a better descriptor. This approach goes beyond identifying high data rate locations and focuses on areas with unexpected declines in data rate, which are crucial for autonomous vehicle operations.

Many insights were acquired from the conducted simulations; for instance, it was learned that vertical curves would have an impact on the scene complexity where the safety risks increase. This is due to occlusions at crest curve locations where the AV becomes hindered or by increasing the number of captured points at sag curves. Further, horizontal curves were found to have a much bigger impact on the performance of the AVs as the occlusion problem is worse at such locations which, again, can jeopardize the operations of the AVs. As for the road layout, it was revealed via the analysis that adding an extra lane will increase the processing requirements for the AVs, which shows that wider roads will naturally be more complex for AVs. Moreover, the weather conditions were found to be a major factor in dictating the complexity of AVs as inclement weather conditions can significantly affect the processing requirements for AVs, which could limit the operations of AVs to favorable weather conditions only to mitigate the safety risks. Finally, the arduous task of handling WVCs can prove to be infeasible for the current AV models, seeing that it massively increases the data rate requirements, making it one of the most challenging tasks for AV operations as it requires the processing of the entire environment instead of focusing on the pertinent road features typically required for navigation.

### 5.1. Volumetric Method

The volumetric method is unique in its approach and exploits the spherical coordinate system used for voxelization. This method assigns more weight to objects with larger volumes, directing its attention to distant points rather than the points near the sensor.

Regarding the analysis of the vertical curves, the volumetric method managed to heighten the effects of the occlusions in both the RFFs and FE road layouts, where they yielded typical behaviors. The percentage of increase in data rates at the sag curve was 42%, 20%, and 18.8%, respectively. The decrease at the crest locations was reported to be 40%, 18.7%, and 22.5%, respectively, for the three road sections. The higher grades of road section one increased the disparity between values on the curves and the flat road sections of the road, as evident in the recorded change percentage in the data rate values. Finally, on comparing the data rate requirements between the FE and RRF analysis in the volumetric method, a gigantic increase was observed due to the presence of roadside features, which prompted the percentages to reach 1300%, 1950%, and 1475%, respectively among the three road sections.

Moving on to the roadway width analysis, the previously established pattern found in the occupancy method calculations was again observed in this approach. Even more so, it was seen in both the RRF and FE layouts. In the RRFs, the 3-lane zones exhibited a 20% increase in the data rate requirements. Whereas, in the FE analysis, the increase percentage was 30%, with the visible influence of vegetation on the results. For the road sections involved in the analysis, the increase in data rate requirements between the RRF and FE analysis ranged between 600 and 700%, significantly lower than that recorded in the vertical curve road sections, due to the overall lower vegetation density witnessed in these sections. Lastly, in the horizontal curve analysis of the modified sections, naturally, the pattern persisted but with much more significant variations in the values owing to the presence of the roadside elements. The drop in data rate requirements reached 491% and 367% at the exit and the entrance of the curves of road sections (8–9), respectively.

**Figure 38:**
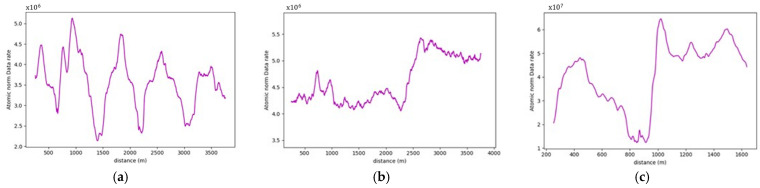
Data rate requirements graphs for volumetric method: (**a**) vertical curves of road section (1); (**b**) roadway width of road section (4); (**c**) horizontal curves of road section (8).

As evident from the results of the volumetric method calculations shown in Figure 38, it becomes apparent that this method is more compatible with RRFs road layouts rather than FE ones. Since it is susceptible to changes in the point densities and point distributions, the presence of roadside features severely affects its results. Generally, the AV will be more concerned with far road points than it would with the vegetation found on its periphery. Hence, it will be less than suitable to use this method to explain the AVs’ performance if the offroad elements are included in the analysis. It, nevertheless, provides robust results in the RRF analysis as it explains, for example, in the vertical curves, the loss of road information at crest curves due to the limited range of vision or the increase in data rate requirements at sag locations owing to the augmented vision at this location. The horizontal curve analysis is a prime example of the shortcomings of the volumetric method, where it reports exaggerated percentages of loss of road information owing to the huge influence of roadside elements. As a result, the volumetric method is not well suited for the analysis of the horizontal curve.

### 5.2. Statical Testing

To validate that the variation in the data rate requirements around the curves and at locations of varying roadway width was not due to chance or from random variables within the environment, a statistical two-sample T-Test was conducted. For such analysis, the null hypothesis is that the mean data rate values do not change at vertical curves, horizontal curves, or varying roadway width locations. Whereas the alternative hypothesis is that there is a variation in the data rate values at such locations. First, the *t*-statistic is calculated, and by knowing the degrees of freedom and significance level, the critical *p*-value can be estimated. The null hypothesis will be rejected if the absolute value of the *t*-statistic is greater than the critical *p*-value. The test results are summarized in Table 6, Table 7 and Table 8.

For the vertical curve analysis, rejecting the null hypothesis would require the variations in the values between the flat and curved locations to be statistically significant on the 99% confidence level. By conducting this analysis on road section (2), the *p*-values for both crest vs. flat and sag vs. flat scenarios were below the *t*-statistic. Consequently, the null hypothesis was dismissed, indicating that there are indeed statistically significant variations in data rate values at the vertical curve locations.

Furthermore, the road was segmented into straight sections and portions corresponding to the horizontal curve in the horizontal curve analysis. Upon analysis, the observed *p*-value was again below the significance threshold, leading to rejecting the null hypothesis. This result indicates a 99% confidence level that the average values at the horizontal curve significantly differ from those outside, thus underscoring the impact of horizontal curve obstructions, distinct from the surrounding environment, on both data rate requirements and the vehicle’s overall performance. Similar results were again seen in the test conducted on the roadway width analysis for section (5) between the 2-lane and 3-lane zones.

## 6. Conclusions

Integrating AVs is a pivotal advancement in the ever-evolving transportation landscape, promising unparalleled safety, efficiency, and accessibility. Understanding the interactions between the AVs and the dynamic physical environment is paramount for ensuring their safety. This study delves into the static surroundings of AVs, aiming to unravel complex scenarios they might encounter. Typically, AVs disregard off-road areas beyond the road shoulders, as these regions do not disrupt their navigational abilities or emergency stops. These irrelevant areas are filtered out from sensor data to streamline processing and reduce computational loads, allowing the vehicle to concentrate on pertinent road features. However, particular challenges, such as wildlife–vehicle collisions (WVCs), demand a holistic approach. Therefore, the analysis is conducted twice: first, by incorporating all roadside features, and second, by excluding them. This dual approach provides a comprehensive understanding of the individual impact of each element on the environment’s complexity. It also sheds light on the additional computational burdens incurred by processing the entire environment, offering invaluable insights into optimizing AV operations.

This research has developed a framework leveraging LiDAR point cloud data from various highways in Alberta, Canada, to create a virtual testing environment. Specifically, this analysis covered 34 km of diverse highway sections, concentrating on two-way, two-lane rural roads. These sections were chosen strategically to represent varied terrains, including flat, rolling, and mountainous landscapes, aiming to model a range of driving environments.

The core principle guiding this analysis is the understanding that the complexity of the environment is directly related to the volume of data that AVs must process. This complexity can be captured by first voxelating the point cloud data surrounding the AV and then calculating the percentage of occupied voxels using the parameter delta (∆). The captured scenes are replicated from the vehicle’s perspective along its trajectory via its sensors using the Vista simulator to ensure accuracy and reliability. Finally, assessing the complexity of the environment is performed using two distinct approaches: the occupancy and volumetric approaches.

This paper’s primary contribution is the development of a framework that can identify and quantify the extent of complexity for the critical locations within the physical infrastructure of AV operations through a transformative function that converts the complexity of the surrounding environment into interpretable real-time data processing needs.

Throughout this analysis, it became apparent that vertical curve occlusion significantly influences the complexity of the environment by affecting the calculated data rate requirements. Across all analyzed sections, a consistent trend emerged, showcasing an increase in scene complexity by up to 7% for sag curves. Additionally, crest locations were identified as areas of heightened complexity, quantified at 4.2%. These regions pose challenges to AVs due to the potential loss of road information. Moreover, critical horizontal curves characterized by tight radii, especially when obstructed by off-road elements like encroaching vegetation, exacerbate occlusion issues. The results underscored the substantial impact of horizontal curve occlusion, with observed increases in scene complexity of up to 19%.

Similarly, an increase of up to 16.5% in scene complexity for AVs was observed by adding an extra lane to the road layout. Notably, weather conditions were identified as a significant factor influencing AV performance, showcasing an inverse relationship that could potentially elevate scene complexity by up to 240%. Roadside features exacerbate the problem of real-time processing as they heavily add to the computational requirements of the AVs if WVCs are to be avoided. This was starkly evidenced in the analysis of full environments (FE), revealing a 400% increase in scene complexity. Finally, the perception of the environment highly depends on the sensors used by the AVs and can jeopardize their operations if the equipped sensor has limited range and precision specifications.

The statistically significant findings in this paper offer insights to AV developers, enhancing their comprehension of how the driving environment impacts AV functions. Government agencies and infrastructure owners and operators (IOOs) can derive significant benefits from this research by evaluating the preparedness of existing infrastructure for AV deployment. Currently, road networks are designed for human-operated vehicles, necessitating thorough reassessments before AV deployment [64]. Furthermore, the acquired insights shed light on the impact of road geometry, roadside features, and roadway width on AV performance. This knowledge is crucial for informing future road designs and ensuring they are optimized for AV usage. Additionally, the simulation environment developed in this work can be used as testing grounds for AV development, where the data processing requirements can be optimized along with the sensor capabilities.

To summarize, the presented analysis indicates that simplifying the surrounding environment, such as reducing the number of lanes, results in a less complex environment for AVs. Additionally, lowering the density of roadside elements, such as environments with open fields, is recommended to enhance AV operations and minimize challenges related to wildlife detection. Furthermore, decreasing road grades and eliminating roadside obstructions at horizontal curves emerge as potent strategies to accommodate AVs effectively. Last but not least, the simulations revealed that weaker sensors can massively affect the perception capabilities of AVs. Hence, it is essential to equip sensors with superior specifications to enhance AV navigation.

While the presented framework is a robust tool for understanding the interaction between AVs and their surrounding environment and anticipating complexity levels in various road and weather conditions, it has a number of limitations. One of the limitations lies in the assumptions endorsed by the implemented algorithm, which might not be universally applicable to all AV models. However, the methodology’s flexibility allows for integrating more advanced and exhaustive algorithms, offering a deeper understanding of AV performance and environmental complexity. Additionally, validating the framework’s results will require the deployment of an AV in the field, which is both costly and has safety risks.

For future endeavors, this framework can be modified to incorporate other sensory information such as cameras and radars by incorporating their specifications in the simulations. Furthermore, a more holistic approach is necessary to comprehensively model the intricacies of dynamic agent interactions with AVs, especially in urban environments where such dynamic objects abound. Additionally, parametric models can be developed to accurately quantify and predict the influence of the different vertical grades and the radii of the horizontal curve on the data rate requirements.

This framework can be further enhanced by adopting a more nuanced approach where the input sensor specification variables can be assumed to follow a probability distribution instead of using deterministic values. Moreover, modeling different sensor heights can also be conducted in the future to assess how it can impact the environment perception and, consequently, the data rate requirements of the AVs.

Last but not least, this work can also be expanded to perform network-level studies, where the most critical locations can be identified to facilitate the optimization process of assigning roadside sensors that can relay real-time information for the AVs on the road to assist them in mitigating and overcoming potentially critical situations.

## Figures and Tables

**Table 1:** LiDAR sensors specifications.

Sensor	R	$\theta_{h}$	$\theta_{l}$	$\phi_{h}$	$\phi_{l}$	$\delta_{R}$	$\delta_{\phi }$	$\delta_{\theta }$	F
VLS-128	245 m	180°	−180°	15	−25°	0.03 m	0.11°	0.11°	20 hz
HDL-32E	100 m	180	−180°	10.7°	−30.7°	0.02 m	1.33°	0.11°	20 hz

**Table 2:** Summary of change in data rate requirements for vertical curves RRF analysis.

Section	Min Value (Crest)	Average Value (Flat)	Max Value (Sag)	% Change Relative to Flat Values
1	$1.01\times 10^{8}$	$1.05\times 10^{8}$	$1.104\times 10^{8}$	−3.9% → +5.1%
2	$0.98\times 10^{8}$	$1.01\times 10^{8}$	$1.04\times 10^{8}$	−3% → +3%
3	$0.96\times 10^{8}$	$1.0\times 10^{8}$	$1.07\times 10^{8}$	−4.2% → +7%

**Table 3:** Summary of RRF data requirements for roadway width analysis.

Section	2-Lane Zone			3-Lane Zone			% Increase
	Min	Max	Average	Min	Max	Average	
4	$1.26\times 10^{8}$	$1.35\times 10^{8}$	$1.3\times 10^{8}$	$1.43\times 10^{8}$	$1.49\times 10^{8}$	$1.46\times 10^{8}$	12.3%
5	$1.10\times 10^{8}$	$1.2\times 10^{8}$	$1.1\times 10^{8}$	$1.29\times 10^{8}$	$1.39\times 10^{8}$	$1.34\times 10^{8}$	16.5%
6	$1.15\times 10^{8}$	$1.25\times 10^{8}$	$1.20\times 10^{8}$	$1.35\times 10^{8}$	$1.40\times 10^{8}$	$1.38\times 10^{8}$	15%
7	$1.15\times 10^{8}$	$1.25\times 10^{7}$	$1.20\times 10^{8}$	$1.34\times 10^{8}$	$1.39\times 10^{8}$	$1.37\times 10^{8}$	14.1%

**Table 4:** Summary for change in data rate values at horizontal curves.

Section	Hz Curve Entrance			Hz Curve Exit		
	Min Value	Max Value	Average Drop	Min Value	Max Value	Average Drop
8	$2.1\times 10^{8}$	$2.5\times 10^{8}$	14%	$2.1\times 10^{8}$	$2.4\times 10^{8}$	19%
9	$2.45\times 10^{8}$	$2.75\times 10^{8}$	12.2%	$2.06\times 10^{8}$	$2.32\times 10^{8}$	12.7%
10	$3.2\times 10^{8}$	$3.7\times 10^{8}$	15.6%	$3.7\times 10^{8}$	$4.3\times 10^{8}$	16.2%

**Table 5:** Summary of data rate requirements for FE vs. RRFs.

Section	Features	RRF Max Value	FE Max Value	% Increase
1	Vertical Curves	$1.1\times 10^{8}$	$5.1\times 10^{8}$	363%
2	Vertical Curves	$1.05\times 10^{8}$	$5.25\times 10^{8}$	400%
3	Vertical Curves	$1.08\times 10^{8}$	$5.25\times 10^{8}$	386%
4	Roadway Width	$1.49\times 10^{8}$	$3.4\times 10^{8}$	128%
5	Roadway Width	$1.39\times 10^{8}$	$3.4\times 10^{8}$	145%
6	Roadway Width	$1.42\times 10^{8}$	$3.3\times 10^{8}$	132%
7	Roadway Width	$1.39\times 10^{8}$	$3.45\times 10^{8}$	148%
8	Horizontal Curves	$1.1\times 10^{8}$	$3.3\times 10^{8}$	200%
9	Horizontal Curves	$1.15\times 10^{8}$	$2.86\times 10^{8}$	149%
10	Horizontal Curves	$1.12\times 10^{8}$	$5.4\times 10^{8}$	382%

**Table 6:** T-Test for road section (2).

Location	Crest	Flat		Sag
Number of Samples	2015	2074		1115
Mean Value	$9.70\times 10^{7}$	$9.72\times 10^{7}$		$9.74\times 10^{7}$
Standard Deviation	$1.67\times 10^{6}$	$1.42\times 10^{6}$		$1.38\times 10^{6}$
Absolute *t*-statistic	3.2		9.3	
*p*-Value	$1.3\times 10^{-3}$		0	

**Table 7:** T-Test for road section (5).

Location	2 Lanes	3 Lanes
Number of Samples	1480	2031
Mean Value	$1.16\times 10^{8}$	$1.32\times 10^{8}$
Standard Deviation	$2.11\times 10^{6}$	$2.54\times 10^{6}$
Absolute *t*-statistic	204	
*p*-Value	0	

**Table 8:** T-Test for road section (7).

Location	Straight	Curved
Number of Samples	1001	243
Mean Value	$2.15\times 10^{8}$	$1.94\times 10^{8}$
Standard Deviation	$2.06\times 10^{7}$	$5.95\times 10^{6}$
Absolute *t*-statistic	27.8	
*p*-Value	0	

## Data Availability

The data that support the findings of this study are available from Alberta Transportation. Restrictions apply to the availability of these data, which were used under license for this study. The authors do not have permission to share these data.
